# Morphological Variability amid Genetic Homogeneity and Vice Versa: A Complicated Case with *Humidophila* (Bacillariophyceae) from Tropical Forest Soils of Vietnam with the Description of Four New Species

**DOI:** 10.3390/plants14071069

**Published:** 2025-03-31

**Authors:** Elena Kezlya, Anton Glushchenko, Yevhen Maltsev, Sergei Genkal, Natalia Tseplik, Maxim Kulikovskiy

**Affiliations:** 1 K.A. Timiryazev Institute of Plant Physiology RAS (IPP RAS), 35 Botanicheskaya St., 127276 Moscow, Russia; melosira@mail.ru (E.K.); closterium7@gmail.com (A.G.); ye.maltsev@gmail.com (Y.M.); ntseplik@gmail.com (N.T.); 2 Papanin Institute for Biology of Inland Waters, Russian Academy of Sciences, 152742 Borok, Russia; genkal47@mail.ru

**Keywords:** Cát Tiên national park, molecular analysis, morphology, soil diatoms, phylogeny, genetic markers, cryptic species

## Abstract

A total of 18 *Humidophila* strains isolated from soil samples from Cát Tiên National Park have been studied. Based on morphometric analysis and molecular data for the V4 18S rDNA and *rbc*L regions, we proposed the presence of four new species: *H. vietnamica*, *H. paravietnamica*, *H. cattiensis*, and *H. concava*. This is the first study that provides molecular data for such a large number of *Humidophila* strains. Furthermore, we encountered some *Humidophila* strains with clear morphological differences (which we assigned to several morphotypes) that cannot be separated using the selected genetic markers and cannot be attributed to phenotypic variations in one species; these require further study of their genetic structure. We also observed the opposite case, where in the absence of morphological differences, clear genetic differentiation is shown, which demonstrates the presence of cryptic taxa in our sample. The maximum differences for these strains were observed in the V4 18S rDNA region. Our results show that the effectiveness of commonly used genetic markers V4 18S rDNA and *rbc*L for separating species can vary greatly. Our study highlights the need to research different genetic markers and their use for proper species separation, as well as the genetic diversity of diatoms, and the need for further studies of intra- and interspecific genetic distances.

## 1. Introduction

The genus *Humidophila* (Lange-Bertalot et Werum) R.L. Lowe et al. was established by R.L. Lowe et al. [1]. The species that were originally placed in subgenus *Paradiadesmis* Lange-Bertalot et Le Cohu of the genus *Diadesmis* Kützing were transferred to *Humidophila* [1]. The main morphological feature used for the division of these genera is the structure of the pore apparatus: representatives of *Diadesmis* uniseriate striae consist of poroids closed by a hymen from the internal side, while representatives of *Humidophila* striae consist of a single elongated areola also closed by a hymen from the internal side. Later, the separation of genera was confirmed by molecular data. There are, however, some species of *Humidophila* that have several areolae per stria [2] or externally give the impression of several areolae in a stria [3], while, on the inside of the valve, there is a single areola; the phylogenetic position of these species has not been confirmed. The results of phylogenetic analysis based on the V4 18S rDNA region showed that representatives of *Diadesmis* and *Humidophila* form independent clades with a sufficient degree of certainty [4]. To date, the genetic diversity of *Humidophila* remains poorly studied. A total of 13 sequences belonging to 10 strains and six taxa are deposited in GenBank. Of these, only two taxa have vouchers in the public domain: *Humidophila sceppacuerciae* Kopalová [5] and *Humidophila* sp. KAS1307 [6]. The latter is incorrectly identified. In the scanning electron microscope images presented for this strain in the publication of Kuehnle et al. [6] (p. 506, Figure 2c,d), the striae consist of a row of poroids, indicating that this strain belongs to the genus *Diadesmis*. Data for the other strains remain unpublished, and no images or other metadata are available in the public domain. All of this complicates the analysis and comparison of molecular data for representatives of *Humidophila* and once again emphasizes the need for the availability of images of strains associated with nucleotide sequences deposited in GenBank.

The genus *Humidophila* currently includes 76 accepted species names and one accepted variety [7]. The vast majority of species are small-celled forms with linear to elliptical valves. They prefer moist subaerial habitats and are found in freshwater bodies [1,8]. Some species are distributed worldwide, while others have a restricted range [9]. For example, *H. contenta* (Grunow) Lowe et al., *H. nienta* (J.R. Carter) Lowe et al., and *H. parallela* (Petersen) Furey, Manoylov et Lowe are cosmopolitan, and *H. contenta* is often noted among the abundant or dominant taxa in aerophilic habitats, e.g., in volcanic soils of the Kamchatka Peninsula (Russia) [10], as indicator species of yellow brown soil in Wuhan (central west China) [11], in epiphytic bryophytes on trees in Wuhan (China) [12], and in the terrestrial samples from James Ross Island (Antarctic Peninsula) [13]. However, it should be noted that, recently, Van de Vijver et al. studied the original sample (Delogne sample 97), determined the taxonomic identity of *H. contenta*, and established the lectotype [14]. They discussed the extremely confusing taxonomic history and emphasized that the identifications and occurrence data for *H. contenta* should always be accompanied by a reference to the taxonomic treatment used. Therefore, the distribution of this species needs to be reconsidered.

Despite the small size of valves and poor morphology, a considerable number of new species has been described recently. A relatively high diversity of representatives of the genus was found in the Antarctic region. In a recent study of the community structure of the terrestrial diatoms from Ulu Peninsula (James Ross Island), *Humidophila* was marked as the most species-rich genus (n = 8) [13]. In less than 10 years, eight species new to science have been described from Antarctica [15,16,17].

Recent studies of aerophilic habitats in subtropical and tropical areas have also reported findings and new species of *Humidophila*. So, Yogeshwaran et al. ([18] and references therein) summarized information on members of the genus in the North Eastern Region of India and described a new aerophilic species from Sada Chiru waterfalls, *H. manipurensis* C. Radhakrishnan, M. Yogehswaran, Kociolek et B. Karthick. The authors point out that only a few species of *Humidophila* have been documented for this region. In a study on diatoms in soils of South India [19], *H. pantropica* (Lange-Bertalot) Lowe et al. was one of the common species in the soils of natural forests and plantations, observed during all seasons with an abundance from 13.3% to 56.6%. Six new species of *Humidophila* have been described in subaerial habitats in the karst region of China [20]. A variety of *Humidophila* species was also reported from terrestrial moss in a cloud forest in Malaysia (seven species) [21] and from urban areas in Indonesia (one species) [22]. According to Ferreira et al. [8], only 12 species of this genus have been recorded in Brazil, with the authors finding and describing a new species of *H. piraquarae* P.C. Ferreira et T.V. Ludwig from a moss on a rocky wall of mountain springs in the state of Paraná (subtropics). Argentina is one of the most biogeographically diverse countries in the world, comprising 18 ecoregions. Summarizing the diversity of the *Humidophila* species, Vouilloud et al. [9] indicated that only six species were known prior to their study. They studied specimens collected in aerophilic microhabitats, mainly associated with waterfalls, from a rainforest known as Paranaense rainforest. As a result, the authors added to the taxon list, reported 17 species recorded in Argentina, and described four new species. One new species, *H. caribaea* M. Rybak, Christenhusz & Byng, was recently described from a tropical forest in Guadeloupe [23].

As far as we know, there is no information about the diversity of representatives of the genus in Vietnam (including in soils). It should also be noted that the diversity of diatoms in the soils of Vietnam is practically unstudied. We only found one study investigating the bioactivities of 13 strains of nostocacean cyanobacteria isolated from paddy soil in Vietnam using a polyphasic approach [24]. Overall, according to a recent review by Joseph and Ray [25], who summarized soil algae studies from 1981 to 2023, data on soil algae from North Africa; Central, East, South, and West Asia; Asia Pacific; and Australia remain poorly explored.

This study is a continuation of a series of studies on microalgae and cyanobacteria in tropical forest soils of Cát Tiên National Park (South Vietnam). The work has been ongoing since 2019, with previous studies describing diatom species new to science (*Mayamaea vietnamica* Glushchenko, Kezlya, Kulikovskiy et Kociolek [26], three species of *Placoneis* Mereschkowsky [27,28,29], *Sellaphora terrestris* Glushchenko, Kezlya, Maltsev, and Kulikovskiy [30] and six species of *Pinnularia* Ehrenberg [31]), as well as representatives of cryptophyta [32] and cyanobacteria [33].

In this paper, the morphology and phylogeny of 18 *Humidophila* strains isolated from soil samples collected from four sites in Cát Tiên National Park are analyzed in detail. Based on the analysis of morphometric characters and comparison of molecular data on V4 18S rDNA and *rbc*L regions, we propose four new species: *H. vietnamica* sp. nov., *H. paravietnamica* sp. nov., *H. cattiensis* sp. nov., and *H. concava* sp. nov. To our knowledge, this is the first study in which molecular data are reported for such a large number of *Humidophila* strains.

## 2. Results

**Figure 1:**
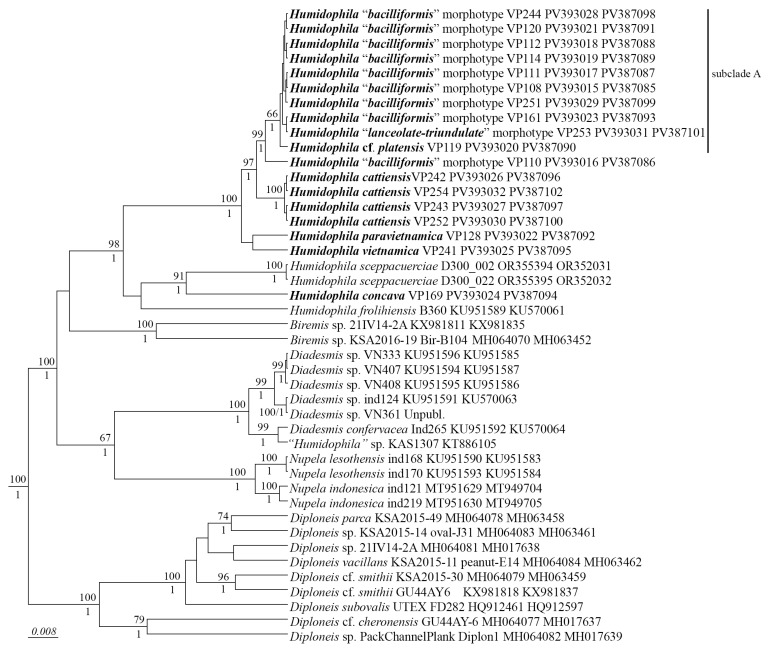
Phylogenetic position of the novel *Humidophila* strains (indicated in bold) based on Bayesian inference for the partial *rbc*L and 18S rRNA genes. The total length of the alignment is 1482 characters. Bootstrap supports (BS) from ML (constructed by RAxML) and posterior probabilities (PP) from BI (constructed by Beast) are presented on the nodes in order. Only BS and PP above 50 and 0.9 are shown. Strain numbers (if available) and GenBank numbers are indicated for all sequences.

We have studied the morphological features of 18 *Humidophila* strains in detail and performed phylogenetic analysis on two marker regions, V4 18S rDNA and *rbc*L. These markers are the most commonly used for diatom phylogenetic analysis [34,35] and have the most data available in GenBank. The phylogenetic analysis shows that all *Humidophila* strains form a single monophyletic clade (Figure 1) with maximum support (BS 98, PP 1.0). Within the clade, some distinct branches corresponding to different species are clearly distinguished (Figure 1). Based on the analysis of morphology and molecular data, we identify four species new to science—*H. cattiensis* sp. nov., *H. vietnamica* sp. nov., *H. paravietnamica* sp. nov., and *H. concava* sp. nov. These species have clear morphological differences and represent separate phylogenetic lineages.

In subclade A consisting of eight strains that are genetically homogeneous by the V4 18S rDNA and *rbc*L regions, we found two well-defined morphotypes: most strains belong to the “*bacilliformis*” morphotype (VP108, VP111, VP112, VP114, VP120, VP161, VP244, and VP251), and one strain (VP253) represents the “*lanceolate-triundulate*” morphotype. Due to the lack of evidence based on molecular analysis, we only provide morphological descriptions and images for these morphotype strains. Since we were not able to identify them accurately, we present a morphology-based comparison with known species.

Strain VP119 represents another morphotype in subclade A, and we identify it as *Humidophila* cf. *platensis* (Metzeltin, Lange-Bertalot et García-Rodríguez) Lowe, Kociolek, Johansen, Van de Vijver, Lange-Bertalot et Kopalová. On the phylogenetic tree, it is somewhat separated from the “*bacilliformis*” and “*lanceolate-triundulate*” morphotype strains. It differs from the latter by 2 bp in the *rbc*L region.

Strain VP110 is clearly genetically separated from the other strains (Figure 1) and forms a separate lineage but morphologically coincides with a number of strains of the “*bacilliformis*” morphotype of subclade A. Due to the lack of sufficient morphological characters, we only give a morphological description for this strain.

**Figure 2:**
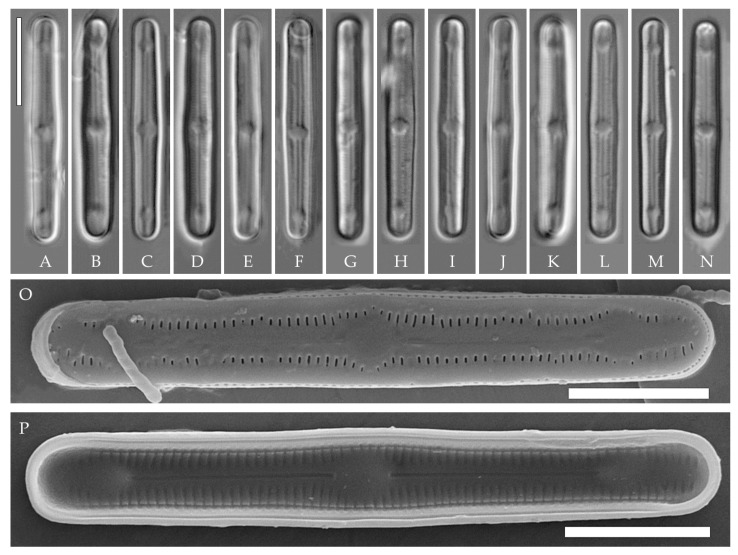
*Humidophila cattiensis* sp. nov. Strain VP242, slide 07000. (**A**–**N**) Light microscopy, differential interference contrast. (**C**) Holotype. Scale bar of 10 μm. (**O**,**P**) Scanning electron microscopy. (**O**) Whole valve, external view. (**P**) Whole valve, internal view. Scale bar of 5 μm.

**Figure 3:**
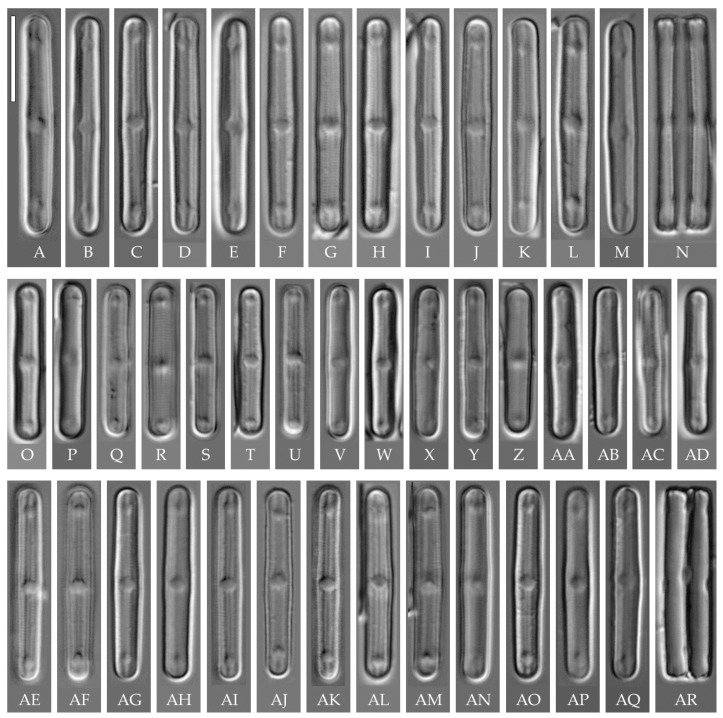
*Humidophila cattiensis* sp. nov. (**A**–**N**) Strain VP243, slide 07001. (**O**–**AD**) Strain VP252, slide 07010. (**AE**–**AR**) Strain VP254, slide 07012. Light microscopy, differential interference contrast. Scale bar of 10 μm. (**A**–**M**,**O**–**AQ**) Valve views. (**N**,**AR**) Girdle views.

**Figure 4:**
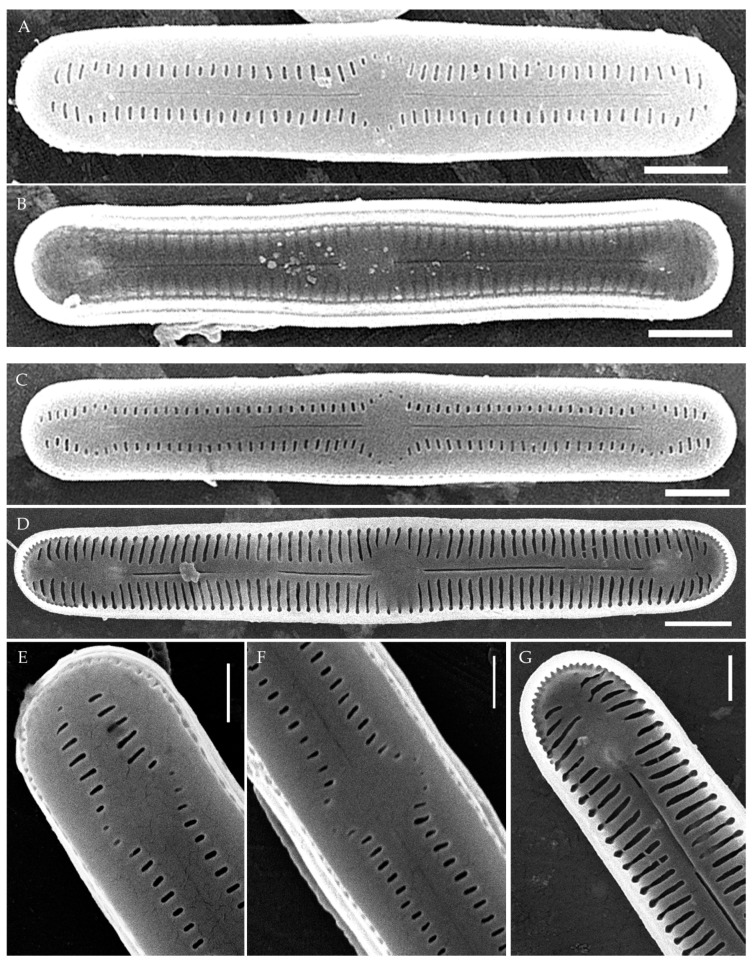
*Humidophila cattiensis* sp. nov. (**A**,**B**) Strain VP252, slide 07010. (**C**–**G**) Strain VP254, slide 07012. Scanning electron microscopy. (**A**,**C**) Whole valve, external view. (**B**,**D**) Whole valve, internal view. (**E**) Valve end, external view. (**F**) Valve center, external view. (**G**) Valve end, internal view. Scale bar of 2 μm (**A**–**D**) and 1 μm (**E**–**G**).

***Humidophila cattiensis* Kezlya, Glushchenko, Maltsev et Kulikovskiy sp. nov. (Figure 2, Figure 3 and Figure 4)**

**Description. LM.** (Figure 2A–N and Figure 3). Valves are linear, very slightly inflated in the middle, with truncated or broadly rounded apices. Valve dimensions (n = 60) are as follows: length of 17–25.5 μm and width of 2.8–3.3 μm. The axial area is narrow and linear, expands in the center to a rounded central area, and also expands near the valve apices to a rounded hyaline area. Fascia is absent. Raphe is straight, filiform, and difficult to distinguish in LM. Striae are not visible in LM.

**SEM** (Figure 2O,P and Figure 4). Externally, striae are parallel throughout the valve or slightly radiate at the valve center (Figure 2O), composed of transapically elongated areolae and located in a longitudinal depression, with a striae density of 30–36 in 10 μm (Figure 2O). Striae terminate near the valve apex. Areolae are shortened or rounded near the central area and the hyaline area at the valve apices. Raphe is straight and filiform, with simple central and terminal endings. The terminal endings do not extend to the edge of the valve and terminate near the hyaline area. The mantle areolae are round and continuous at the valve apices (Figure 2O and Figure 4E).

Internally, areolae are slit-like throughout the valve and shortened around the central area and the hyaline area at the valve apices (Figure 2P and Figure 4D,G). Raphe is filiform, straight, central raphe endings simple, distal endings terminate in poorly developed helictoglossae (Figure 4). Areolae are covered by hymenes (Figure 4B).

**Holotype here designated**: Slide no. 07000a (represented here by Figure 2C), deposited in the Herbarium of K.A. Timiryazev Institute of Plant Physiology, Russian Academy of Sciences (HD), Moscow, Russia, prepared from oxidized culture strain VP242 isolated from sample Kt15.

**Isotype.** Slide no. 07000b, Herbarium of Main Botanical Garden, Russian Academy of Science (MHA), Moscow, Russia.

**Reference strain**. VP242 from the Culture and Barcode Collection of Microalgae and Cyanobacteria “Algabank” (СВМС), isolated from sample no. Kt15.

**Representative specimens**. Strain VP252 (slide no. 07010, sample no. Kt16), VP254 (slide no. 07012, sample no. Kt18), VP243 (slide no. 07001, sample no. Kt15).

**Type locality**. Southeast Vietnam, Cát Tiên National Park, test plot “Ficus” (11°26.112′ N, 107° 25.424′ E), forest soil surface, at depth of 5–10 cm and in the leaf litter.

**Sequence data**. Partial 18S rRNA gene sequences comprising V4 domain (GenBank accession numbers: PV387096 for VP242, PV387097 for VP243, PV387100 for VP252, PV387102 for VP254) and partial *rbc*L sequences (GenBank accession numbers: PV393026 for VP242, PV393027 for VP243, PV393030 for VP252, PV393032 for VP254).

**Etymology**. The specific epithet “*cattiensis*” refers to the name of Cát Tiên National Park where this species was observed.

**Distribution**. So far, the species is only known from the Cát Tiên National Park (in forest soils and in the leaf litter).

We have observed four strains of *H. cattiensis* sp. nov. Morphologically, the strains are similar in valve outline and structure and mostly vary in valve length, with strains VP242 and VP243 exhibiting longer valves (24.6–25.5 μm), strain VP254 exhibiting slightly smaller valves (22.0–22.8 μm), and strain VP252 exhibiting the smallest valves (17.0–18.0 μm).

*H. cattiensis* sp. nov. can be confused with *H. vietnamica* sp. nov. and *H. paravietnamica* sp. nov., which are also described in this study. The species are similar in size and in the presence of hyaline areas at the ends of the valves and striae density (Table 1). *H. cattiensis* sp. nov. can be easily distinguished from the former species by the valve shape—*H. vietnamica* sp. nov. has inflated, spatulate apices, whereas, in *H. cattiensis* sp. nov., the apices are not inflated. *H. vietnamica* sp. nov. is also differentiated by elongated areolae at the valve ends. The differences between *H. cattiensis* sp. nov. and *H. paravietnamica* sp. nov. are subtle—the valves of the latter are slightly narrower (Table 1) and have tapering ends—whereas, in *H. cattiensis* sp. nov., the ends are straight. All three species are clearly separated on the basis of phylogenetic analysis. The tropical species *H. pantropica* differs from the new species by a lower striae density (25–27 in 10 µm in *H. pantropica* vs. 30–36 in 10 µm in the new species), and *H. subtropica* (Metzeltin, Lange-Bertalot et Garcia-Rodriguez) Lowe et al. differs by smaller valves (length of 12–18 µm in *H. subtropica* vs. 17.5–26 in the new species). Also among similar species in shape and size are *H. amsterdamensis* Chattová et Van de Vijver, *H. komarekiana* Kochman-Kędziora, Noga, Zidarova, Kopalová et Van de Vijver and *H. australoshetlandica* Kopalová, Zidarova et Van de Vijver, but *H. cattiensis* sp. nov. is clearly distinguished by the presence of hyaline areas at the valve ends.

**Figure 5:**
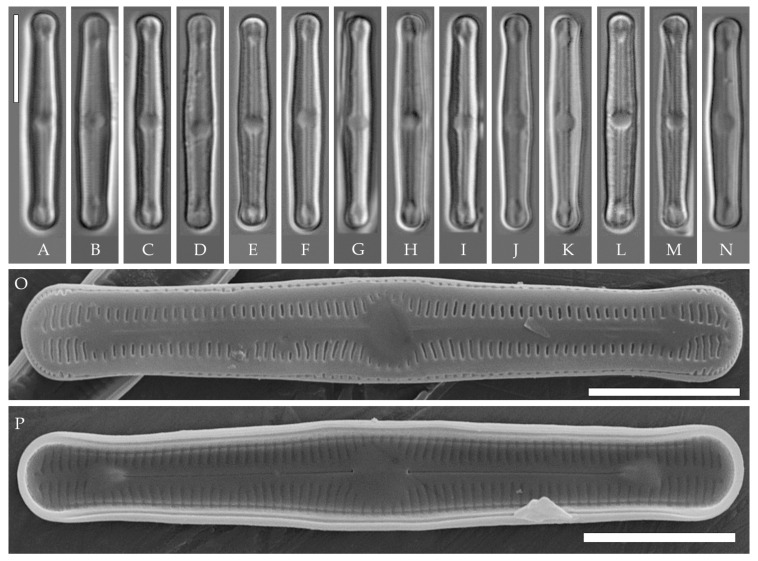
*Humidophila vietnamica* sp. nov. Strain VP241, slide 06999. (**A**–**N**) Light microscopy, differential interference contrast. (**E**) Holotype. Scale bar of 10 μm. (**O**,**P**) Scanning electron microscopy. (**O**) Whole valve, external view. (**P**) Whole valve, internal view. Scale bar of 5 μm.

***Humidophila vietnamica* Kezlya, Glushchenko, Maltsev et Kulikovskiy sp. nov. (Figure 5)**

**Description. LM** (Figure 5A–N). Valves are linear and inflated in the middle. Apices are inflated, spatulated, and broadly rounded. Valve dimensions (n = 20) are as follows: length of 24.5–25.5 μm and width of 3.0–3.5 μm. The axial area is narrow and linear, expands in the valve center to a rounded central area, and also expands near the valve apices to a rounded hyaline area. Raphe is straight and filiform and terminates near the hyaline area. Striae are not resolvable in LM.

**SEM** (Figure 5O,P). Externally, striae parallel throughout the valve or slightly radiate at the valve center (Figure 5O), composed of short transapically elongated or nearly rounded areolae (36 in 10 μm), with the areolae elongated at the apices, located in a longitudinal depression (Figure 5O). Raphe is straight, filiform, with simple, straight central and terminal endings. The terminal endings do not extend to the edge of the valve and terminate near the axial hyaline area. Striae terminate near the valve apex. Mantle areolae are continuous at the valve apices (Figure 5O).

Internally, areolae are slit-like throughout the valve, shortened around the central area and the hyaline area at the valve apices and elongated at the apices (Figure 5P). Raphe is filiform and straight, with simple central raphe endings and distal endings terminating in weakly developed helictoglossae (Figure 5P). Areolae are covered by hymenes (Figure 5P).

**Holotype here designated**: Slide no. 06999a (represented here by Figure 5E), deposited in the Herbarium of K.A. Timiryazev Institute of Plant Physiology, Russian Academy of Sciences (HD), Moscow, Russia, prepared from oxidized culture strain VP241 isolated from the soil sample Kt10.

**Isotype**. Slide no. 06999b, Herbarium of Main Botanical Garden, Russian Academy of Science (MHA), Moscow, Russia.

**Reference strain.** VP241 from the Culture and Barcode Collection of Microalgae and Cyanobacteria “Algabank” (СВМС), isolated from sample no. Kt10.

**Type locality**. Southeast Vietnam, Cát Tiên National Park, test plot “Vyshka” (11° 26.490′ N, 107° 24.063′ E), forest soil, at depth of 3–10 cm.

**Sequence data.** Partial 18S rRNA gene sequences comprising V4 domain (GenBank accession number: PV387095) and partial *rbc*L sequences (GenBank accession number: PV393025).

**Etymology**. The specific epithet refers to the name of the country where this species was observed.

**Distribution**. So far, the species is only known from the Cát Tiên National Park (forest soils).

**Comments**

A comparison of *H. vietnamica* sp. nov. with the closely related species *H. cattiensis* sp. nov. and *H. paravietnamica* sp. nov. described in this study is given above. Similar tropical species to *H. vietnamica* sp. nov. also include *H. pantropica*, reported from Oceania, Southeast Asia, South America, and Hawai’i [37], and *H. subtropica*, found in the lit cave wall samples on the Big Island of Hawai’i. The species are similar in valve shape and the presence of hyaline areas at the apices. The new species can be clearly distinguished from *H. pantropica* by the striae density (clearly distinct in LM, 28–30 in 10 μm in *H. pantropica* vs. 34–36 in 10 μm, not resolvable in LM in *H. vietnamica* sp. nov.). Compared to *H. subtropica*, the new species is similar in valve shape and striae density, but the valves of the new species are larger (length of 25–25.5 μm vs. 12–18 μm in *H. subtropica*). *H. vietnamica* sp. nov. also lacks the central raised structure characteristic for *H. subtropica* [1]. A distinctive feature of the new species is the elongated areolae at the valve apices, while, in *H. subtropica*, the areolae at the valve apices are slightly shortened.

In terms of valve shape and striae density, the new species can be confused with *H. costei* (Le Cohu et Van de Vijver) R.L. Lowe, Kociolek, J.R. Johansen, Van de Vijver, Lange-Bertalot et Kopalová, which was described from Antarctica [38] (p. 220), but the valves of the latter are wider (3.5–4.5 μm vs. 3.0–3.5 μm in *H. vietnamica* sp. nov.) and lack hyaline areas at the apices.

**Figure 6:**
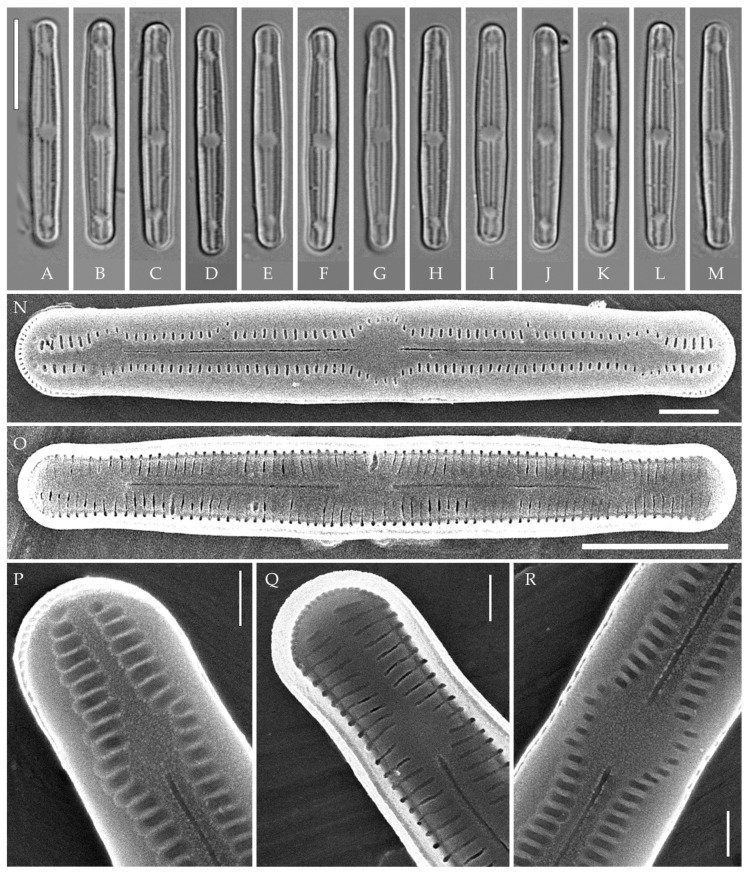
*Humidophila paravietnamica* sp. nov. Strain VP128, slide 06766. (**A**–**M**) Light microscopy, differential interference contrast. (**B**) Holotype. Scale bar of 10 μm. (**N**–**R**) Scanning electron microscopy. (**N**) Whole valve, external view. (**O**) Whole valve, internal view. (**P**) Valve end, external view. (**Q**) Valve end, internal view. (**R**) Valve center, external view. Scale bar of 5 μm (**O**), 2 μm (**N**), and 1 μm (**P**–**R**).

***Humidophila paravietnamica* Kezlya, Glushchenko, Maltsev et Kulikovskiy sp. nov. (Figure 6)**

**Description. LM** (Figure 6A–M). Valves are linear, with slightly narrowed to broadly rounded, sometimes subcapitate, apices. Valve dimensions (n = 20) are as follows: length of 24.5–26.0 μm and width of 2.5–3.0 μm. The axial area is narrow and linear, expands in the center to a rounded central area, and also expands near the valve apices to a rounded hyaline area. Raphe is straight and filiform and terminates near the hyaline area. Striae are not resolvable in LM.

**SEM** (Figure 6N–R). Externally, striae are parallel throughout the valve or slightly radiate at the center (Figure 6N), composed of transapically elongated areolae (33–35 in 10 μm), located in a longitudinal depression (Figure 6P). Areolae are shorter near the central area and at the hyaline area at the valve apices, sometimes elliptic or rounded. Raphe is straight and filiform with simple, straight central and terminal endings. The terminal endings do not extend to the edge of the valve, terminating near the hyaline area. Striae terminate near the valve apex.

Internally, areolae are slit-like throughout the valve, parallel throughout the valve or slightly radiate at the valve center, and shortened around central area and around the hyaline area at the valve apices (Figure 6O,Q). Raphe is filiform and straight, with simple central and terminal endings and terminal endings with poorly developed helictoglossae (Figure 6O,Q). Areolae are covered by hymenes (Figure 6Q).

**Holotype here designated**: Slide no. 06766a (represented here by Figure 6B), deposited in the Herbarium of K.A. Timiryazev Institute of Plant Physiology, Russian Academy of Sciences (HD), Moscow, Russia, prepared from oxidized culture strain VP128 isolated from the sample Kt56.

**Isotype**. Slide no. 06766b, Herbarium of Main Botanical Garden, Russian Academy of Science (MHA), Moscow, Russia.

**Reference strain.** VP128 from the Culture and Barcode Collection of Microalgae and Cyanobacteria “Algabank” (СВМС), isolated from sample no. Kt56.

**Type locality**. Southeast Vietnam, Cát Tiên National Park (11° 26.975′ N, 107° 21.462′ E), basalt on soil surface.

**Sequence data**. Partial 18S rRNA gene sequences comprising V4 domain (GenBank accession number: PV387092) and partial *rbc*L sequences (GenBank accession number: PV393022).

**Etymology**. The specific epithet refers to the new species being close to *Humidophila vietnamica* sp. nov. in phylogenetic position and similar in morphology.

**Distribution**. So far, the species is only known from the Cát Tiên National Park (forest soils).

**Comments**

*Humidophila paravietnamica* sp. nov. can be confused with the phylogenetically close species *H. vietnamica* sp. nov., which is discussed above. The species can be distinguished by the valve shape—in *H. paravietnamica* sp. nov. the ends are narrowed, whereas, in *H. vietnamica* sp. nov., they are clearly widened, and the valves of *H. paravietnamica* sp. nov. are narrower (2.5–3.0 μm vs. 3.0–3.5 μm in *H. vietnamica* sp. nov.). Another difference between these species is the absence of elongated areolae at the apices in *H. paravietnamica* sp. nov. (Figure 6N,P), and the areolae throughout the valve are approximately the same size and shape, whereas, in *H. vietnamica* sp. nov., elongated areolae are present at the apices (Figure 5O).

**Figure 7:**
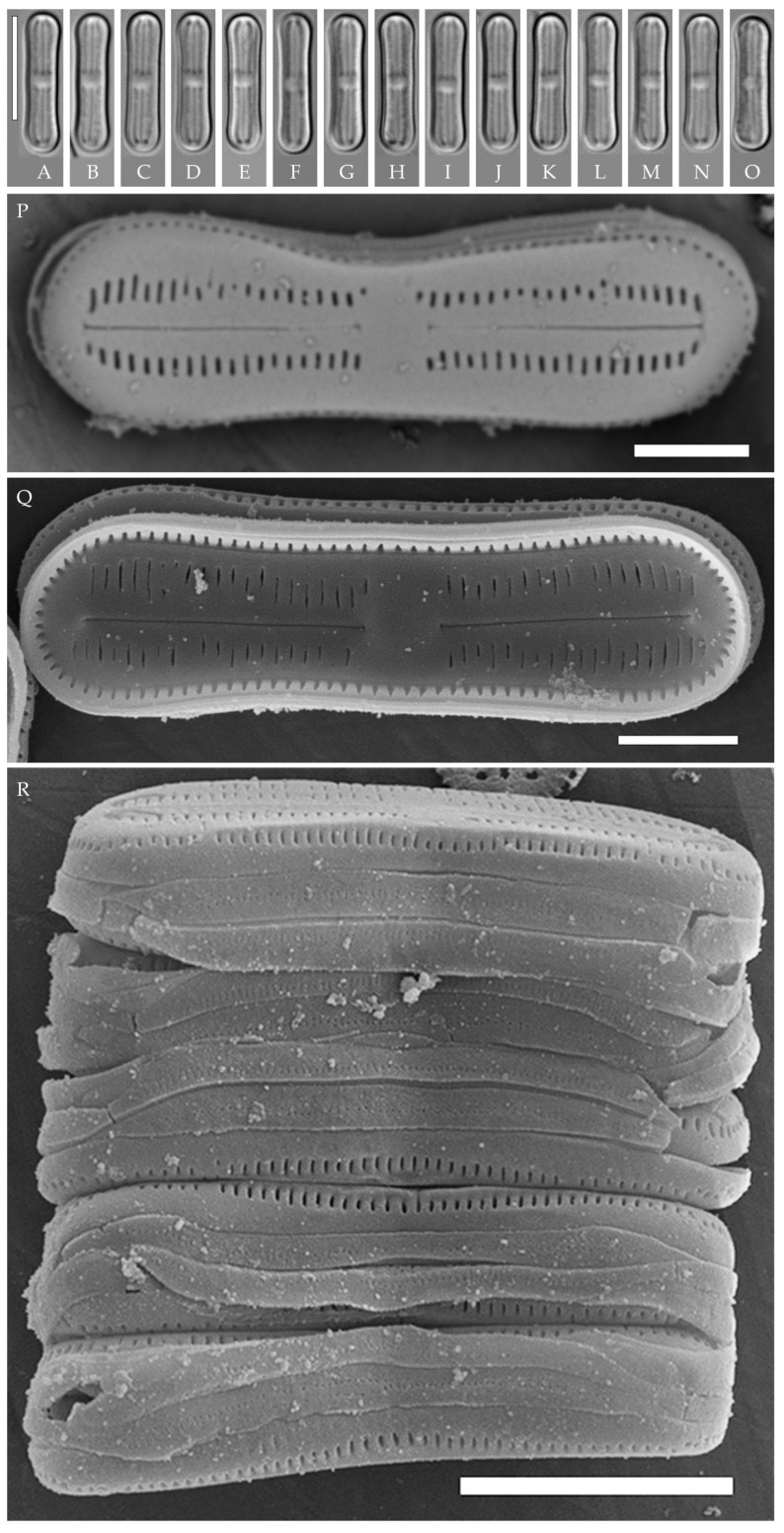
*Humidophila concava* sp. nov. Strain VP169, slide 06852. (**A**–**O**) Light microscopy, differential interference contrast. (**C**) Holotype. Scale bar of 10 μm. (**P**–**R**) Scanning electron microscopy. (**P**) Whole valve, external view. (**Q**) Whole valve, internal view. (**R**) Several valves in girdle view. Scale bar of 2 μm.

***Humidophila concava* Kezlya, Glushchenko, Maltsev et Kulikovskiy sp. nov. (Figure 7)**

**Description. LM** (Figure 7A–O). Valves are small and concave in outline, with swollen, broadly rounded apices. Valve dimensions (n = 20) are as follows: length of 12.8–13.5 μm, width of 2.5–3.0 μm, and apex width of 3.5 μm. The axial area is relatively broad, linear or slightly inflated near the ends, expanding in the valve center to a fascia (sometimes only on one side). Raphe is straight and filiform with simple or T-shaped central and terminal endings. Striae are not resolvable in LM.

**SEM** (Figure 7P–R). Externally, striae are parallel throughout the valve (Figure 7P), composed of transapically elongated or slit-like areolae (40–45 in 10 μm). In the central area, areolae can be absent (fascia), or rounded areolae may be present on only one side (Figure 7P). Raphe is straight and filiform, with simple or T-shaped central endings (Figure 7P). Terminal raphe endings are simple, connected with the last areolae by shallow grooves. Mantle areolae continue around the valve apices, without any interruption (Figure 7P,Q). Girdle bands are open, with one row of rounded poroids (Figure 7R). Cells form ribbon-like colonies (Figure 7R).

Internally, areolae are slit-like, parallel throughout the valve, absent on the central area (Figure 7Q). Raphe filiform, straight, raphe endings simple, terminal raphe endings terminate in weakly developed helictoglossae (Figure 7Q).

**Holotype here designated**: Slide no. 06752a (represented here by Figure 7C), deposited in the Herbarium of K.A. Timiryazev Institute of Plant Physiology, Russian Academy of Sciences (HD), Moscow, Russia, prepared from oxidized culture strain VP169 isolated from the sample Kt53.

**Isotype**. Slide no. 06752b, Herbarium of Main Botanical Garden, Russian Academy of Science (MHA), Moscow, Russia.

**Reference strain.** VP169 from the Culture and Barcode Collection of Microalgae and Cyanobacteria “Algabank” (СВМС), isolated from sample no. Kt53.

**Type locality**. Southeast Vietnam, Cát Tiên National Park (11° 26.975′ N, 107° 21.462′ E), basalt on soil surface.

**Sequence data**. Partial 18S rRNA gene sequences comprising V4 domain (GenBank accession number: PV387094) and partial *rbc*L sequences (GenBank accession number: PV393024).

**Etymology**. The specific epithet refers to the valve outline with concave margins.

**Distribution**. So far, the species is only known from the Cát Tiên National Park (forest soils).

**Comments**

Currently, there are at least 10 species very similar in size and valve shape (concave in outline, with swollen, broadly rounded apices) to *H. concava* sp. nov. (Table 2). The species are distinguished on the basis of small differences that can only be identified in detailed SEM studies. The closely related tropical species *H. manipurensis* described from Manipur, India, differs from the new species by a higher striae density (50 in 10 µm vs. 40–45 in 10 µm in *H. concava* sp. nov.) and the presence of shallow grooves on both sides of the proximal and distal raphe ends. In the new species, the distal raphe ends are connected with the last areolae by shallow grooves, whereas, near the proximal raphe ends, the shallow grooves are absent. Also, in *H. manipurensis*, externally, areolae are small and rounded, whereas, in *H. concava* sp. nov., they are slit-like.

From *H. misionera* Vouilloud, Guerrero et Kociolek described from a waterfall in Argentina, the new species differs by having a higher striae density (40–45 in 10 µm vs. 34–36 in 10 µm in *H. misionera*) and a different structure of terminal raphe ends—in *H. misionera*, they are simple and occasionally flanked by small, shallow depressions, whereas, in the new species, they are T-shaped, connected with the last areolae by shallow grooves. *H. lagartiensis* Vouilloud, Guerrero et Kociolek was also described from the waterfall in Argentina. The central raphe endings are different (T-shaped in *H. lagartiensis* vs. simple in the new species; distal raphe ends are flanked by troughs, whereas, in the new species, they are T-shaped and connected with the last areolae by shallow grooves. Also, in *H. lagartiensis*, externally, areolae are small and rounded, whereas, in *H. concava* sp. nov., they are slit-like.

*H. parallela* is smaller in length (7.2–9.1 µm vs. 12.8–13.5 µm in *H. concava* sp. nov.) and has a significantly lower striae density (34–34.9 µm vs. 40–45 in *H. concava* sp. nov.), and the T-shaped depression of the proximal raphe ends is absent.

*H. paracontenta* var. *magisconcava* (Lange-Bertalot) R.L. Lowe et al. has a lower striae density [39] (Figures 60–64) (28–30 µm vs. 40–45 in *H. concava* sp. nov.), with very short comma-like depressions flanking the central and terminal raphe ends (instead of T-shaped terminal endings in *H. concava* sp. nov.). It is worth noting that in the illustrations of *H. paracontenta* var. *magisconcava* presented by Lange–Bertalot and Werum (Figures 60–64, [39]), it can be seen that the fascia is developed inconsistently—present (Figures 61 and 62, [39]), absent (Figures 60 and 63, [39]), and one-sided (Figure 64, [39]). This feature is observed in the new species as well (Figure 7).

*H. biscutella* (Gerd Moser, Lange-Bertalot et Metzeltin) Lowe, Kociolek, Johansen, Van de Vijver, Lange-Bertalot et Kopalová [40] (p. 260, Figures 1–4 and 10) has mantle areolae that are interrupted at the apices (vs. not interrupted in *H. concava* sp. nov.), and the central and terminal raphe ends are without any depressions, while shallow grooves connect terminal raphe ends with the last areolae in *H. concava* sp. nov. Also, in *H. biscutella*, the striae density is lower (36–40 in 10 µm vs. 40–45 in 10 µm in *H. concava* sp. nov.).

*H. delognei* Goeyers et Van de Vijver differs from the new species by valve outline (linear with parallel margins and non-protracted apices vs. concave, with swollen, broadly rounded apices in the new species), as well as central and terminal raphe endings terminating in a shallow T- to Y-shaped groove, while only terminal raphe endings do so in *H. concava* sp. nov.

*H. tahitiensis* (Lange-Bertalot et Werum) Lowe, Kociolek, Johansen, Van de Vijver, Lange-Bertalot et Kopalová has central and terminal raphe endings that terminate in a laterally expanded depression (distinct T-shaped fissures, as deeper narrow incisions running into one or two pairs of the areolae), this feature is absent in *H. concava* sp. nov.

*H. simplex* (E. Reichardt) R.L. Lowe et al. has shorter striae with rounded, almost never transapically elongated areolae vs. the slit-like areolae in the new species, T-shaped central and terminal raphe endings vs. only terminal raphe endings in *H. concava* sp. nov., as well as a lower striae density (34–38 in 10 µm vs. 40–45 in 10 µm, respectively).

*H. discordabilis* (Gerd Moser, Lange-Bertalot et Metzeltin) Lowe, Kociolek, Johansen, Van de Vijver, Lange-Bertalot et Kopalová is larger than *H. concava* sp. nov. (see Table 2), externally, the areolae are short and rounded (vs. slit-like in the new species), and there are no T-shaped depressions at the terminal raphe ends that are characteristic for the new species.

*H. deceptionensis* Kopalová, Zidarova et Van de Vijver has lower striae density (resolvable in LM, 30–32 in 10 µm vs. 40–45 in 10 µm in *H. concava* sp. nov.) and large mantle areolae that are clearly interrupted near the valve apices vs. continuous in the new species. The depressions at the raphe ends are absent.

**Table 2:** Main morphometric features of *Humidophila concava* sp. nov. and similar taxa.

Taxon (Strain)	Length (µm)	Width (µm)	Striae Density in 10 µm	Features	Reference
*H. concava* sp. nov. (VP169)	12.8–13.5	2.5–3.0 (apex width of 3.5 μm)	40–45	Slit-like areolae	this study
*H. manipurensis*	9.5–13.0	2.0–3.0	50	Shallow grooves present on both sides of the proximal and distal raphe ends; internally, a well–developed central nodule present at center of the valve; rectangular fascia	[18]
*H. misionera*	8.8–11.7	2.3–3.0	34–36	Very distinct fascia; terminal raphe endings simple and occasionally flanked by small, shallow depressions	[9]
*H. lagartiensis*	8.2–10.8	2.9–3.5	38–40	Externally, central raphe endings are T-shaped, terminal raphe endings are straight; troughs are present	[9]
*H. parallela*	7.2–9.1	2.0–3.1	34–34.9	T-shaped depression of the proximal raphe ends absent	[41]
*H. paracontenta* var*. magisconcava*	7–13	2.0–3.3	28–30	Very short comma-like depressions flanking central and terminal pores of the raphe	[39]
*H. biscutella*	8–12	2.0–2.5	36–40	Without any depressions near raphe ends; mantle areolae are interrupted at the apices	[40]
*H. delognei*	7–13	2.5–3.0	40	Valves linear with parallel margins and non-protracted apices; central and terminal raphe endings terminating with shallow T- to Y-shaped grooves; central nodule clearly raised	[42]
*H. tahitiensis*	11.2–14.0 8.5–11.0	2.9–3.8 3.0–3.4	37–42	Proximal and distal raphe endings terminating in a laterally expanded depression, often intersecting the areolae (T-shaped fissures)	[1,39]
*H. simplex*	6.0–11.5	2.1–2.9 (apex width of 3.5 μm)	34–38	Clear rectangular fascia; shorter striae with rounded, almost never transapically elongated areolae	[14,43] (p. 432)
*H. deceptionensis*	9.0–12.5	2.7–3.1	resolvable in LM 30–32	Mantle areolae clearly interrupted near the valve apices; depressions at the raphe ends are absent	[15]
*H. discordabilis*	16–20	5.5–6.5	28–32	Depressions at the raphe ends are absent	[40] (Tafel 27, p. 258)

**Figure 8:**
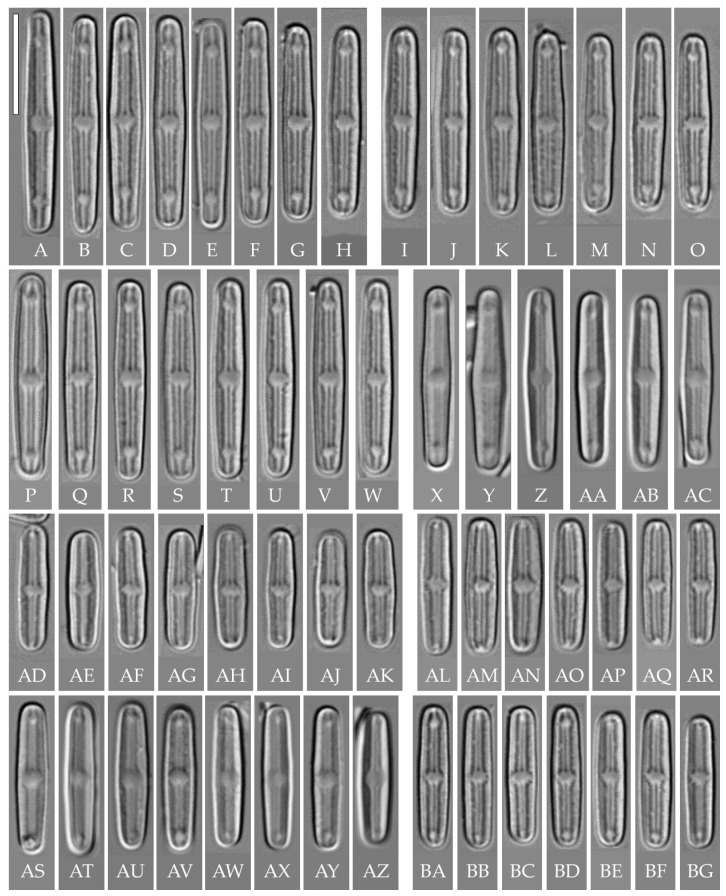
*Humidophila* “*bacilliformis*” morphotype. (**A**–**H**) Strain VP108, slide 06771. (**I**–**O**) Strain VP114, slide 06763. (**P**–**W**) Strain VP112, slide 06775. (**X**–**AC**) Strain VP251, slide 07009. (**AD**–**AK**) Strain VP120, slide 06756. (**AL**–**AR**) Strain VP161, slide 06844. (**AS**–**AZ**) Strain VP244, slide 07002. (**BA**–**BG**) Strain VP111, slide 06774. Light microscopy, differential interference contrast. Scale bar of 10 μm.

**Figure 9:**
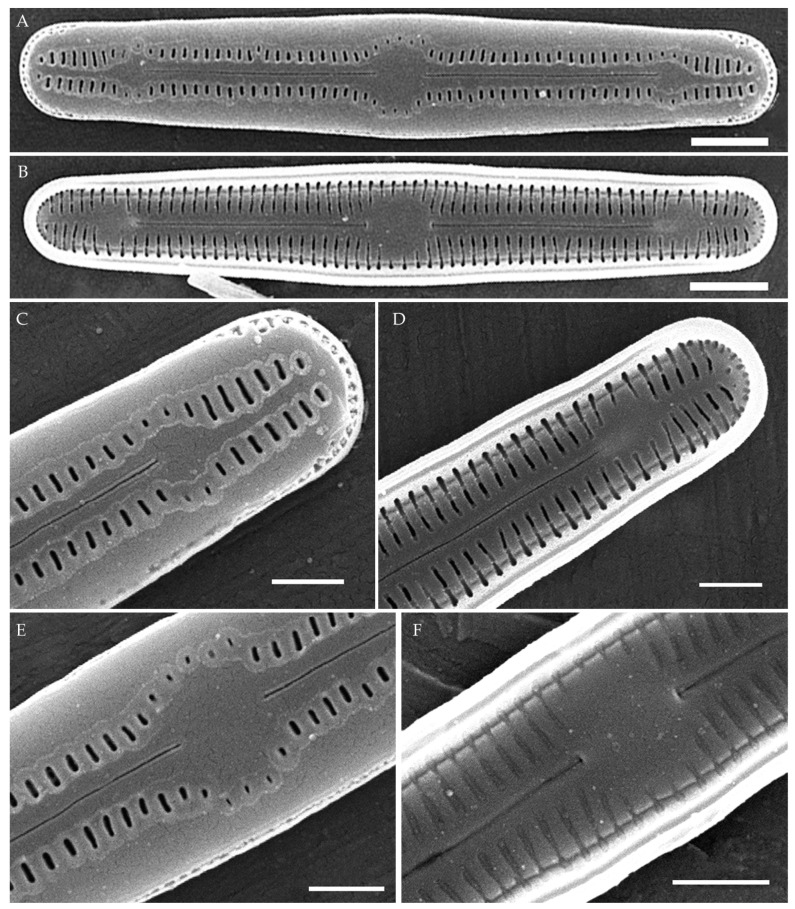
*Humidophila* “*bacilliformis*” morphotype. Strain VP108, slide 06771. Scanning electron microscopy. (**A**) Whole valve, external view. (**B**) Whole valve, internal view. (**C**) Valve end, external view. (**D**) Valve end, internal view. (**E**) Valve center, external view. (**F**) Valve center, internal view. Scale bar of 2 μm (**A**,**B**) and 1 μm (**C**–**F**).

**Figure 10:**
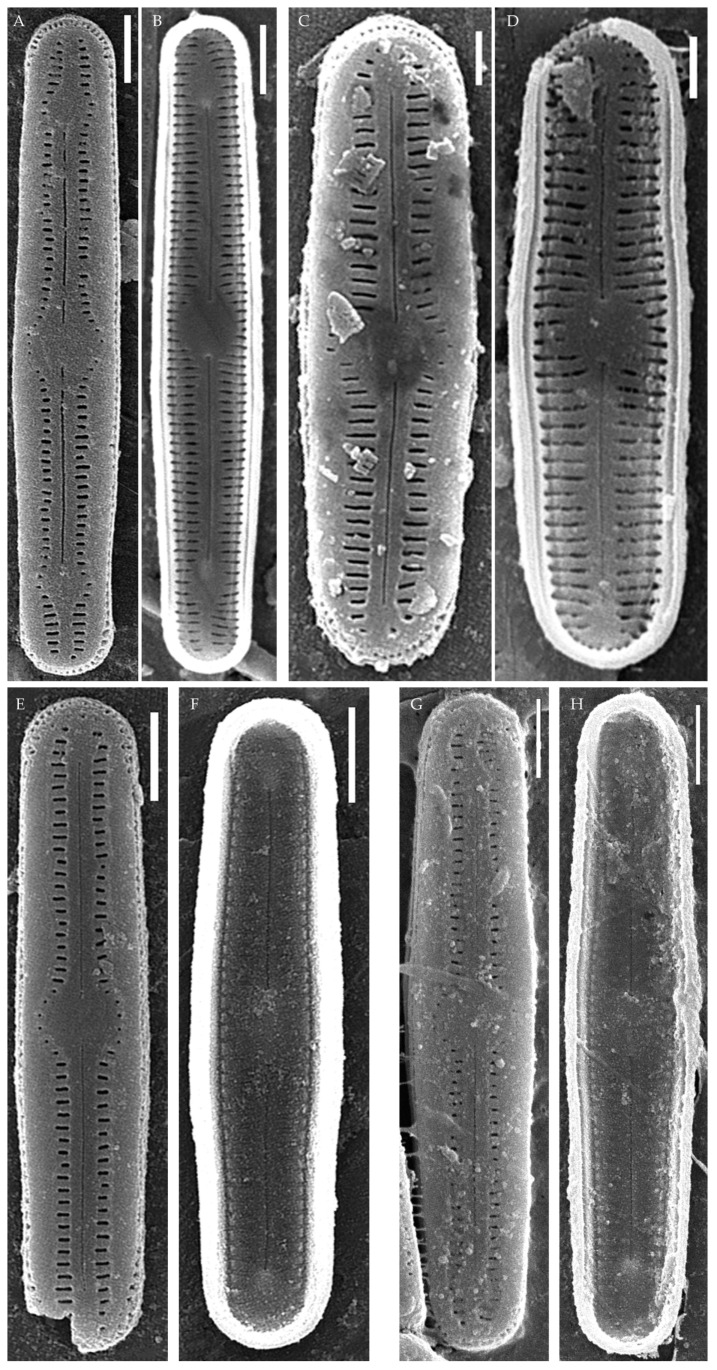
*Humidophila* “*bacilliformis*” morphotype. Scanning electron microscopy. (**A**,**B**) Strain VP112, slide 06775. (**C**,**D**) Strain VP120, slide 06756. (**E**,**F**) Strain VP244, slide 07002. (**G**,**H**) Strain VP251, slide 07009. (**A**,**C**,**E**,**G**) Whole valve, external view. (**B**,**D**,**F**,**H**) Whole valve, internal view. Scale bar of 2 μm (**A**,**B**,**E**–**H**) and 1 μm (**C**,**D**).

**Figure 11:**
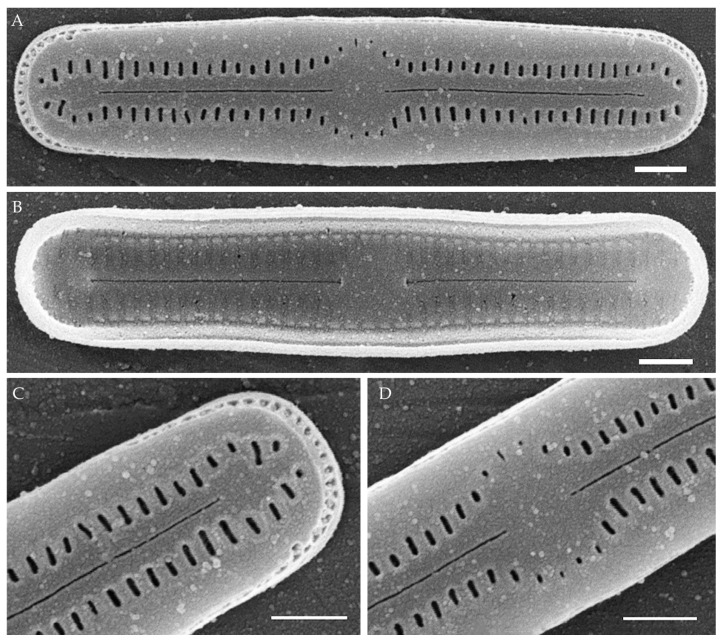
*Humidophila* “*bacilliformis*” morphotype. Strain VP111, slide 06774. Scanning electron microscopy. (**A**) Whole valve, external view. (**B**) Whole valve, internal view. (**C**) Valve end, external view. (**D**) Valve center, external view. Scale bar of 1 μm.

***Humidophila*** **“*bacilliformis*” morphotype (subclade A) (strains VP108, VP111, VP112, VP114, VP120, VP161, VP244, VP251) (Figure 8, Figure 9, Figure 10 and Figure 11)**

**General formal description**. **LM** (Figure 8). Valves are linear in outline and slightly widened in the middle, with apices broadly rounded. Valve dimensions (n = 50 on average for each strain) of the strains are presented in Table 3: length range of 8.5–22.0 μm and width of 2.5–3.2 μm. The axial area is narrow and linear, expands in the valve center to a rounded central area, and also expands near the valve apices to a rounded hyaline area. The hyaline areas are absent in strain VP120; they may not always be present in smaller valves or may be developed on only one side (strain VP120 (Figure 8AD–AK and Figure 10C,D)). Raphe is straight, filiform, and difficult to distinguish in LM. Fascia is absent. Striae are not resolvable in LM.

**SEM** (Figure 9, Figure 10 and Figure 11). Externally, striae are parallel throughout the valve or slightly radiate at the valve center, composed of transapically elongated areolae, located in a longitudinal depression. Striae density ranges from 33–36 (VP112, VP114) to 36–40 in 10 µm (VP108, VP111, VP120, VP244, and VP251) (Figure 8, Figure 9, Figure 10 and Figure 11). Areolae are elliptic or rounded near the central area and the hyaline area at the valve apices. Raphe is straight and filiform, with simple, straight central and terminal endings. The terminal endings do not extend to the edge of the valve, terminating near the axial hyaline area. Striae terminate near the valve apex. Mantle areolae are continuous at the valve apices (Figure 9A,C, Figure 10A,C,E and Figure 11A,C).

Internally, areolae are slit-like throughout the valve and shortened around the central area and around the hyaline area at the valve apices (Figure 9B,D,F, Figure 10B,D,F and Figure 11B). Raphe is filiform, straight, with T-shaped central endings, simple terminal endings, with and weakly developed helictoglossae (Figure 9B,D,F, Figure 10B,D,F and Figure 11B). Areolae covered by hymenes (Figure 9B,F, Figure 10F and Figure 11B).

Slides from oxidized culture strains deposited in the Herbarium of K.A. Timiryazev Institute of Plant Physiology, Russian Academy of Sciences (HD), Moscow, Russia. For sequence data, no. of the slides, and type locality, see the “Materials and Methods” section.

Strain VP161 was established from the soil surface sample Kt26 from the test plots “Afzelia”, strains VP108, VP111, VP112, VP114, and VP120 were established from the soil surface sample Kt9 from the test plots “Vyshka”, and strains VP244 and VP251 were established from the soil sample Kt16 (depth 5–10 cm) from the test plots “Ficus”.

**Comments**

All eight strains assigned by us to the “*bacilliformis*” morphotype (subclade A) have the same linear valve shape with broadly rounded apices, rounded central margin, simple raphe ends, areolae located in depressions on the outer surface of the valve, and the same valve width range (2.5–3.0 μm). The strains differ among themselves only in valve length and a slight variation in striae density (Table 3). The length of small-celled strains (VP111, VP161, VP120, and VP244) ranges from 11.5 to 16.0 µm, and the length of larger-celled strains (VP108, VP112, VP114, and VP251) ranges from 18.0 to 22.0 µm.

Small-celled strains of the “*bacilliformis*” morphotype (VP111, VP161, VP120, and VP244) are very close in size, valve shape, and ultrastructure to *H. comperei* (Le Cohu et Van de Vijver) Lowe, Kociolek, Johansen, Van de Vijver, Lange-Bertalot et Kopalová, described from lakes in the Kerguelen Archipelago in the subantarctic [44], and to the tropical *H. iguazuensis* Guerrero, Vouilloud et Sala described from a waterfall in Argentina [9] and *H. lacunosa* (Gerd Moser, Lange-Bertalot et Metzeltin) Lowe, Kociolek, Johansen, Van de Vijver, Lange-Bertalot et Kopalová from New Caledonia. However, in *H. comperei* and *H. lacunose*, the raphe almost reaches the end of the valve and ends at the level of the last stria [44] (Figures 22 and 23), [44] (Figure 3A–E); in *H. iguazuensis*, the raphe ends are bordered by irregular troughs that extend laterally and intersect with the last striae [9] (Figures 25–27). On the valves of our strains (Figure 10C,E), the raphe does not reach the end of the valve, terminating at the level of 3–4 striae from the end of the valve (hyaline area undeveloped), and irregular troughs are absent.

Among the species close to strains of the “*bacilliformis*” morphotype, we should note *H. vojtajarosikii* Kopalová, Zidarova et Van de Vijver, *H. keiliorum* Kopalová [15] and *H. komarekiana* [16]. These species clearly differ from our strains by the interruption in the mantle areolae at the apices (in strains of the “*bacilliformis*” morphotype, the mantle areolae at the apices are continuous) and lower striae density (27–32 in 10 µm in the three species vs. 33–40 in 10 µm in strains of the “*bacilliformis*” morphotype) (Table 3). When comparing LM images, strains of the “*bacilliformis*” morphotype can also be confused with *H. virginiana* (Lange-Bertalot) Lowe, Kociolek, Johansen, Van de Vijver, Lange-Bertalot et Kopalová) [39]. *H. “bacilliformis”* can be easily distinguished from the latter by the presence of hyaline areas at the ends of the valves. Also distinctive in *H. virginiana* are the circumpolar areolae that are apically elongated and continue onto the valve face from the mantle [39] (Figures 100–105).

Strains VP108, VP112, VP114, and VP251 with valve length from 18.0 to 22.0 µm are similar to *H. komarekiana* and *H. keiliorum* Kopalová. However, the valves of these three species are wider (>3 µm vs. 2.5–3.0 in strains of *H. “bacilliformis”*), the striae density is lower (27–32 in 10 µm vs. 33–40 in 10 µm in *H. “bacilliformis”*), hyaline areas at the apices are absent, and a clear interruption in mantle areolae is present.

**Figure 12:**
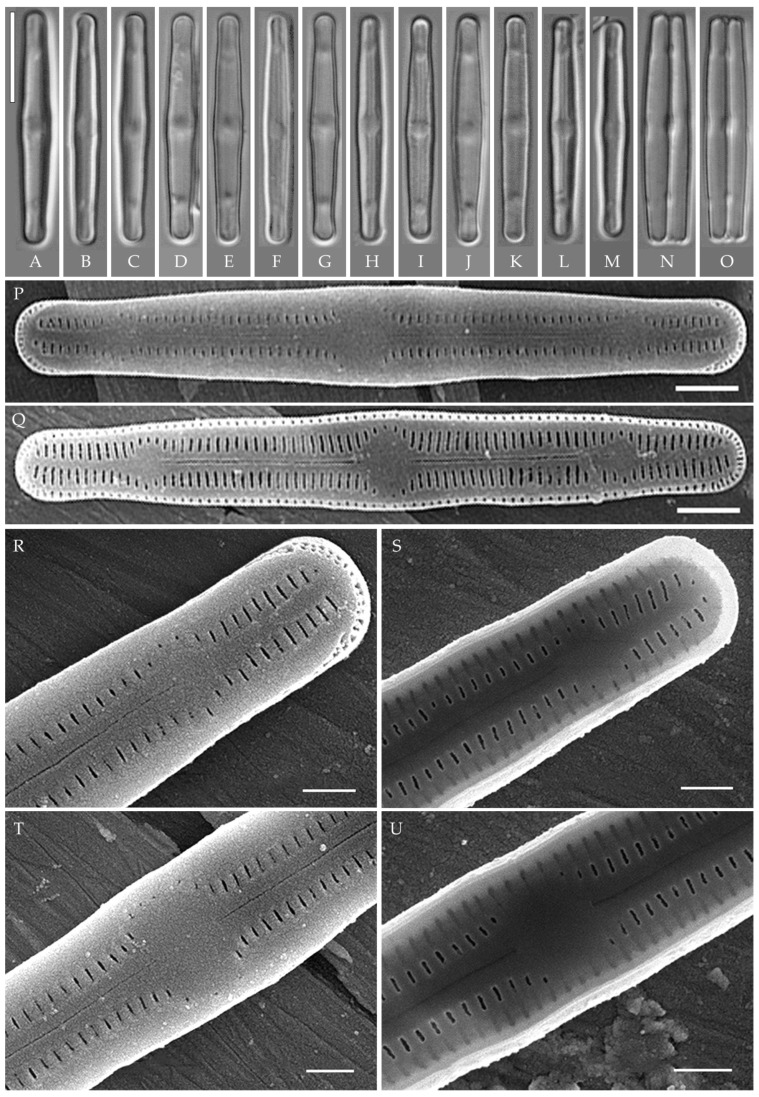
*Humidophila* “*lanceolate-triundulate*” morphotype. Strain VP253, slide 07011. (**A**–**O**) Light microscopy, differential interference contrast. (**A**–**M**) Valve views. (**N**,**O**) Girdle views. Scale bar 10 μm. (**P**–**U**) Scanning electron microscopy. (**P**) Whole valve, external view. (**Q**) Whole valve, internal view. (**R**) Valve end, external view. (**S**) Valve end, internal view. (**T**) Valve center, external view. (**U**) Valve center, internal view. Scale bar of 2 μm (**P**,**Q**) and 1 μm (**R**–**U**).

***Humidophila “lanceolate-triundulate”* morphotype (strain VP253) (Figure 12)**

**Formal description. LM** (Figure 12A–O). Valves are linear-lanceolate in outline, slightly triundulate, with apices narrow, spatulated, broadly rounded, and not capitated. Valve dimensions (n = 20) are as follows: length of 24.5–26.2 μm, width of 2.5–3.2 μm, and apex width of 2.0–2.1 μm. The axial area is narrow and linear, expands in the valve center to a rounded central area, and also expands near the valve apices to a rounded hyaline area. Raphe is straight, filiform, and difficult to resolve in LM. Fascia is absent. Striae are not resolvable in LM.

**SEM** (Figure 12P–U). Externally, striae are parallel throughout the valve or slightly radiate at the valve center (Figure 12P), composed of transapically elongated areolae (34–36 in 10 μm), at the apices located in a longitudinal depression (Figure 12R). Areolae are slit-like, shorter, or rounded near the central area and at the hyaline area at the valve apices. Raphe is straight and filiform, with simple, straight central and terminal endings. The terminal endings do not extend to the end of the valve, terminating near the hyaline area. Striae terminate near the valve apex. Mantle areolae are continuous at the valve apices (Figure 12R).

Internally, areolae are slit-like throughout the valve, shortened around the central area and around the hyaline area at the valve apices (Figure 12S,U). Raphe is filiform and straight and has simple central endings and terminal endings with very weakly developed helictoglossae (Figure 12S,U). Areolae are covered by hymenes.

Slide no. 07011 from oxidized culture strain VP253 deposited in Herbarium of K.A. Timiryazev Institute of Plant Physiology, Russian Academy of Sciences (HD), Moscow, Russia. For sequence data and type locality, see “Materials and Methods”. Strain VP253 was established from the soil sample Kt16 (depth 5–10 cm) from the test plots “Ficus”.

**Comments**

*H. “lanceolate-triundulate”* morphotype clearly differs from the other strains of the clade by the shape of the valve (inflated in the middle and narrowed protracted ends). The valves are longer compared to the “*bacilliformis*” morphotype (24.5–26.5 µm vs. 11.5–22.0) (Figure 8 and Figure 12).

In valve outline, *H. “lanceolate-triundulate”* resembles *H. aspera* Goeyers et Van de Vijver [42], but the former is slightly longer (24.4–26.5 µm vs. 8.0–18 µm in *H. aspera*), has lower striae density (34–36 in 10 µm vs. 40–45 in 10 µm in *H. aspera*), and has clearly protracted ends (Table 4). Also, *H. aspera* differs by the presence of irregular siliceous thickenings in the axial area and the central area and the presence of mantle areolae that are located on the valve near the apices, which are absent in *H. “lanceolate-triundulate”*.

Very subtle differences in valve outline can be observed between *H. “lanceolate-triundulate”* and *H. paravietnamica* sp. nov. *H. “lanceolate-triundulate”* differs by having a triundulate valve outline and more narrowed, spatulate apices (Figure 12); in *H. paravietnamica* sp. nov., the valves are linear and very slightly concave in the middle with narrowed broadly rounded apices (Figure 6).

**Figure 13:**
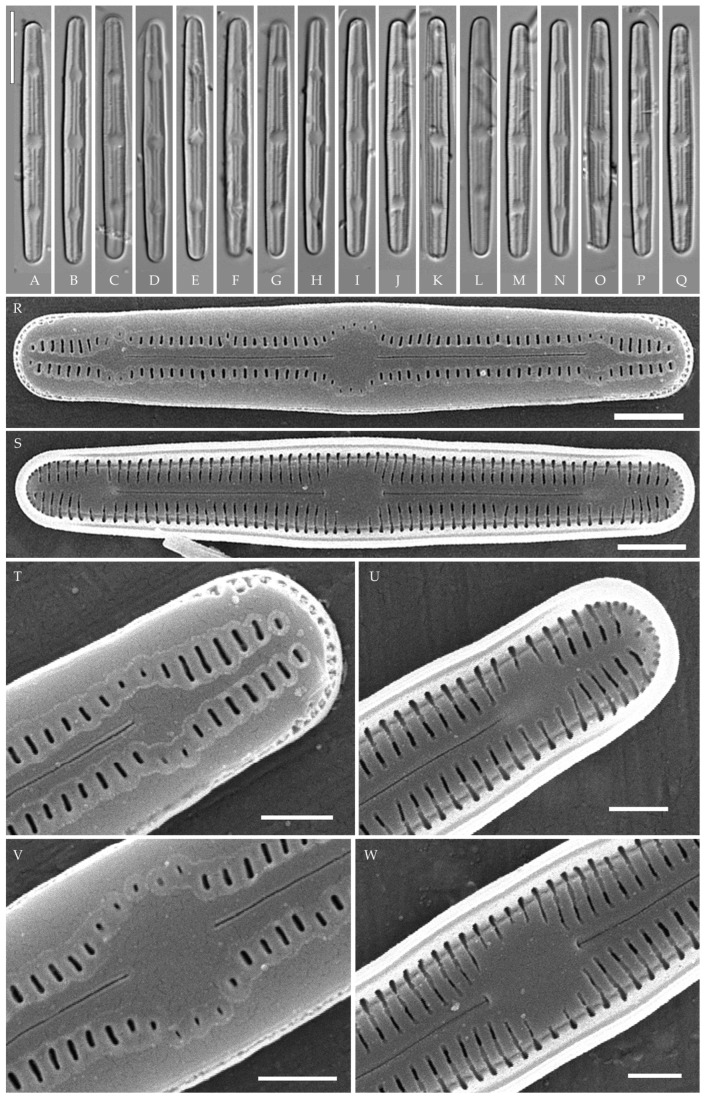
*Humidophila* cf. *platensis*. Strain VP119, slide 06751. (**A**–**Q**) Light microscopy, differential interfer-ence contrast. Scale bar 10 μm. (**R**–**W**) Scanning electron microscopy. (**R**) Whole valve, external view. (**S**) Whole valve, internal view. (**T**) Valve end, external view. (**U**) Valve end, internal view. (**V**) Valve center, external view. (**W**) Valve center, internal view. Scale bar of 2 μm (**R**,**S**) and 1 μm (**T**–**W**).

***Humidophila*** **cf. *platensis* (Metzeltin, Lange-Bertalot et García-Rodríguez) Lowe, Kociolek, Johansen, Van de Vijver, Lange-Bertalot et Kopalová (strain VP119) (Figure 13** **)**

**Formal description. LM** (Figure 13A–Q). Valves are linear, slightly narrowed to the broadly rounded apices. Valve dimensions (n = 20) are as follows: length of 32.0–35.0 μm and width of 2.8–3.2 μm. The axial area is narrow and linear, expands in the valve center to a rounded central area, and also expands near the valve apices to a rounded hyaline area. Raphe is straight and filiform and terminates near the hyaline area. Striae are not resolvable in LM.

**SEM** (Figure 13R–W). Externally, striae are parallel throughout the valve and slightly radiate at the valve center (Figure 13R), composed of transapically elongated slit-like areolae located in a longitudinal depression (36 in 10 μm) (Figure 13T). Areolae are shorter, elliptic, or rounded near the central area and near the hyaline area at the valve apices. Raphe is straight and filiform, with simple, straight central and terminal endings. The terminal endings do not extend to the end of the valve, terminating near the hyaline area. Striae terminate near the valve apex. Mantle areolae are continuous at the valve apices (Figure 13T).

Internally, areolae are slit-like throughout the valve, shortened around the central area and around the hyaline area at the valve apices (Figure 13S). Raphe is filiform and straight, with simple central raphe endings and terminal endings with weakly developed helictoglossae (Figure 13U,W). Areolae are covered by hymenes (Figure 13U,W).

Slide no. 06751 from oxidized culture strain VP119 deposited in Herbarium of K.A. Timiryazev Institute of Plant Physiology, Russian Academy of Sciences (HD), Moscow, Russia. For sequence data and type locality, see “Materials and Methods”. Strain VP119 was established from the soil surface sample Kt9 from the test plots “Vyshka”.

**Comments**

Strain VP119 is morphologically and ultrastructurally very close to *H. platensis* (Metzeltin, Lange-Bertalot et García-Rodríguez) Lowe, Kociolek, Johansen, Van de Vijver, Lange-Bertalot et Kopalová described from Uruguay (South America). All features correspond, including the outline, length, striae density, presence of hyaline areas at the apices, and areolae located in a longitudinal depression [36] (p. 41 and p. 357, plate 56, Figures 1–10). There are only slight differences from the type description in cell width (2.8–3.2 µm in strain VP119 vs. 3.5 in *H. platensis*). We, therefore, identify it as *H.* cf. *platensis*. Other similar species include *H. potapovae* Lowe, Kociolek et You, *H*. sp. [20] (Figures 27–38), *H. elegans* (Moser, Lange-Bertalot et Metzeltin) Lowe, Kociolek, Johansen, Van de Vijver, Lange-Bertalot et Kopalová, *H. irata* (Krasske) Lowe, Kociolek, Johansen, Van de Vijver, Lange-Bertalot et Kopalová. These clearly differ by lower striae density (27–30 in 10 µm vs. 35–36 in 10 µm in *H. platensis* and strain VP119) and in some other properties as listed in Table 5.

*H.* sp. sensu Lowe et al. 2017 (Figures 27–38, [20])

**Figure 14:**
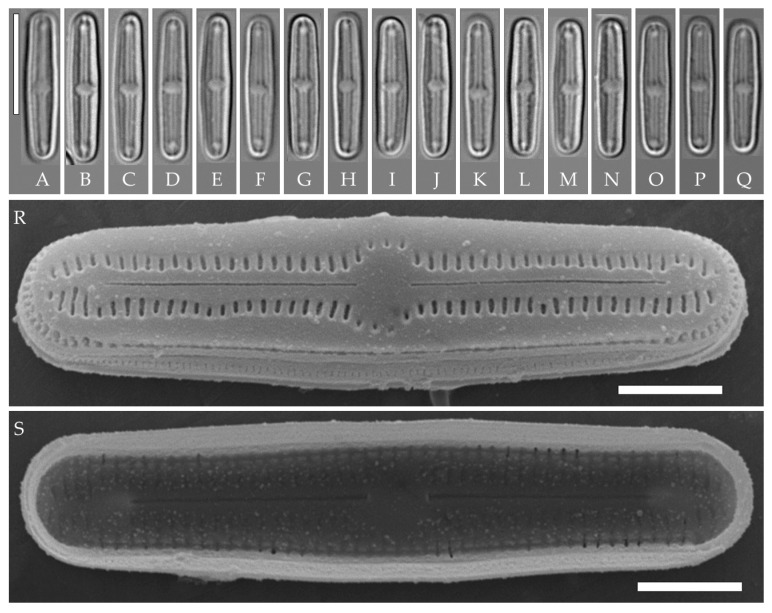
*Humidophila* “*bacilliformis*” morphotype. Strain VP110, slide 06769. (**A**–**Q**) Light microscopy, differential interference contrast. Scale bar of 10 μm. (**R**,**S**) Scanning electron microscopy. (**R**) Whole valve, external view. (**S**) Whole valve, internal view. Scale bar of 2 μm (**R**,**S**).

***Humidophila* “*bacilliformis*” morphotype (strain VP110) (Figure 14)**

**Table 3:** Main morphometric features of *H*. “*bacilliformis*” morphotype strains and similar taxa.

Taxon (Strain)	Length (µm)	Width (µm)	Striae Density in 10 µm	Features	Reference
*H. “bacilliformis”* (VP161)	12.5–14.0	2.5–2.9	n.d.	Hyaline areas at the apices absent	this study (Figure 8AL–AR)
*H. “bacilliformis”* (VP114)	18.0–19.0	2.5–3.0	34–36	ALD *; hyaline areas at the apices present	this study (Figure 8I–O)
*H. “bacilliformis”* (VP112)	19.0–20.5	2.5–3.0	33–34	ALD; hyaline areas at the apices present	this study (Figure 8Q–X and Figure 10A,B)
*H. “bacilliformis”* (VP108)	20.0–22.0	2.5–3.0	36.5–40	ALD; hyaline areas at the apices present	this study (Figure 8A–H and Figure 9)
*H. “bacilliformis”* (VP251)	17.5–19.0	2.3–3.0	36–38	ALD; hyaline areas at the apices present	this study (Figure 8Y–AC)
*H. “bacilliformis”* (VP120)	11.5–12.7	2.5–3.0	38–40	ALD; hyaline areas difficult to distinguish in LM, adjacent to valves apices	this study (Figure 8AD–AK and Figure 10C,D)
*H. “bacilliformis”* (VP244)	14.0–16.0	2.3–3.0	36–36.5	ALD; hyaline areas at the apices are present	this study (Figure 8AS–AZ and Figure 10E,F)
*H. “bacilliformis”* (VP111)	13.8–14.5	2.5–3.0	38–40	ALD; hyaline area absent or present on one or both apices	this study (Figure 8BA–BG and Figure 11)
*H. “bacilliformis”* (VP110)	14.5–16.0	2.5–3.0	35	ALD	this study (Figure 14)
*H. comperei*	6.0–15.0 (type) 9.1–13.3	2.0–3.0 2.2–3.1	30–34 31–36	Hyaline areas at the apices absent	[44,45]
*H. vojtajarosikii*	7.5–12.5	2.5–3.0	30–32	Mantle areolae clearly interrupted near the valve apices; hyaline areas at the apices absent	[15]
*H. virginiana*	16.0–20.0	2.8–3.1	38–40	Circumpolar areolae apically more or less elongated and continue onto the valve face from the mantle; hyaline areas at the apices absent	[39]
*H. iguazuensis*	5.5–16.0	2.5–3.0	36–40	Hyaline areas at the apices absent	[9]
*H. lacunosa*	10.0–15.0	2.8–3.5	36	Cuneate rather than rounded apices; hyaline areas at the apices absent	[40]
*H. komarekiana*	14.0–20.0	3.0–4.0	29–32	Mantle areolae clearly interrupted near the valve apices; hyaline areas at the apices absent	[16]
*H. keiliorum*	10.4–31.2	3.2–5.2	27–30	Mantle areolae clearly interrupted near the valve apices; hyaline areas at the apices absent	[15] (p. 123); [30]

* ALD—areolae located in a longitudinal depression.

**Table 4:** Main morphometric features of *H. “lanceolate-triundulate”* and similar taxa.

Taxon (Strain)	Length (µm)	Width (µm)	Striae Density in 10 µm	Features	Reference
*H. “lanceolate-triundulate”* (VP253)	24.4–26.5	2.7–3.2	34–36	Lanceolate, triundulate valve outline; ALD only at apices	this study
*H. paravietnamica* sp. nov. (VP128)	24.5–26.0	2.5–3.0 (apex width of 2.3–2.7)	33–35	ALD	this study
*H. aspera*	8.0–18.0	2.5–3.0	40–45	Valve surface uneven, covered by irregular siliceous thickenings; central area with a distinct rounded thickening; mantle striae at the apices located on the valve face	[42]

**Table 5:** Main morphometric features of *Humidophila* cf. *platensis* (strain VP119) and similar taxa.

Taxon (Strain)	Length (µm)	Width (µm)	Striae Density in 10 µm	Features	Reference
*H.* cf. *platensis* (VP119)	32.0–35.0	2.8–3.2	36	ALD	this study
*H. platensis*	30.0–33.0	3.5	35–36	ALD	[36] (p. 356)
*H. potapovae*	15.5–27.0	3.0–3.5	30–32	Axial area shows shallow indented depressions	[20]
*H.* sp. sensu Lowe et al. 2017 (Figures 27–38, [20])	26.0–48.0	3.4–3.8	29–30	ALD, nodules, and raphe on elevated axial area	[20]
*H. elegans*	20.0–30.0	4.0–5.0	27–30	—	[40] (p. 144, pl. 29, Figure 7)
*H. irata*	30.0–33.0	3.5	30	Irregular pattern of circular depressions on the valve face	(p.114); [1,20,39,46]

Strain VP110 is morphologically similar to other strains of the “*bacilliformis*” morphotype that were discussed above, thus, we assign it to the same morphotype; however, on the phylogenetic tree, it forms a branch that is clearly separated from subclade A (Figure 1), which includes the other “*bacilliformis*” strains. Therefore, we find it appropriate to discuss strain VP110 separately.

**Formal description. LM** (Figure 14A–Q). Valves are linear with broadly rounded apices. Valve dimensions (n = 20) are as follows: length of 14.5–16.0 μm and width of 2.5–3.0 μm. The axial area is narrow and linear, expands in the valve center to a rounded central area, and also very slightly expands near the valve apices to a small hyaline area. Raphe is straight and filiform and terminates near the hyaline area. Striae are not resolvable in LM.

**SEM** (Figure 14R–S). Externally, striae are parallel throughout the valve or slightly radiate at the valve center (Figure 14R), composed of transapically elongated areolae (35 in 10 μm), located in a longitudinal depression (Figure 14R). Areolae are shorter to rounded near the central area and slightly shorter near the hyaline area at the valve apices (Figure 14R). Raphe is straight and filiform, with simple, straight central and terminal endings. Terminal raphe endings do not extend to the end of the valve, terminating near 2–5 striae from the valve end or near the hyaline area. Striae terminate near the valve apex. Mantle areolae are continuous at the valve apices (Figure 14R).

Internally, areolae are slit-like throughout the valve, parallel throughout the valve or slightly radiate at the valve center, shortened around the central area (Figure 14S). Raphe is filiform and straight, with T-shaped central endings and terminal endings with weakly raised helictoglossae (Figure 14S). Areolae are covered by hymenes (Figure 14S).

Representative specimen. Slide no. 06769, from oxidized culture strain VP110, isolated from the forest soil sample Kt9 (see Materials and Methods), deposited in the Herbarium of K.A. Timiryazev Institute of Plant Physiology, Russian Academy of Sciences (HD), Moscow, Russia. Found only in sample Kt9.

## 3. Discussion

### 3.1. Phylogenetic Position of Humidophila

Presently, the genus *Humidophila* together with *Diadesmis* is assigned to the family Diadesmidaceae (suborder Neidiineae). However, molecular studies have repeatedly shown that these genera are not sister to each other; representatives of *Humidophila* form an independent clade unrelated to *Diadesmis*. At the same time, the exact phylogenetic position of *Humidophila* has not yet been determined. In previous studies based on 18S rRNA gene sequences, representatives of *Humidophila* occupied a position next to *Berkeleya* Greville and *Frustulia* Rabenhorst [4], and based on two *rbc*L and partial 18S rRNA genes, next to *Amphora* Ehrenberg ex Kützing and *Halamphora* (Cleve) Mereschkowsky [47]. In our study, *Humidophila* occupies a place next to members of the genus *Biremis* D.G. Mann et E.J. Cox (Figure 1). In all cases, there is no support for the clades of the tree, which indicates an unstable position and the need for further study of the phylogenetic position of representatives of this genus.

### 3.2. Combined Analysis of Genetic and Morphometric Differences Between Studied Humidophila Strains

A number of papers devoted to the targeted study of diatom species complexes with discussion of genetic distance report that the sequence divergence of particular *rbc*L and 18S rDNA regions between closely related species is small. So, Hamsher et al. [48] studied the discriminate power of four DNA barcode markers for diatoms (*rbc*L; *rbc*L-3P; 28S rDNA D2/D3; and UPA, as well as COI-5P.T) using the example of closely related species of the *Sellaphora* Mereschkowsky complex. The authors revealed that the lowest divergence between species pairs is 0.14% (only 2 bp) for *rbc*L. Generally, the species were divergent by 0.4–7.3% (6–104 bp). Sequence divergence for *rbc*L-3P was only 0.1–0.3% (1–2 bp) for the most closely related species and 0.6–10.6% (4–74 bp) divergent for the remaining species of the *Sellaphora* complex. The authors note that, while the sequence divergence for *rbc*L marker is low, there were no species with identical sequences.

Vanormelingen et al. [49] studied in detail the morphology and genetic variability of small-celled *Sellaphora* strains from the United Kingdom and Australia based on cox1 and *rbc*L genetic regions. Analyzing the intraspecific and interspecific variability of the genetic marker *rbc*L, the authors note that the intraspecific variability is small (0–2 bp or 0–0.16% and up to 6 bp or 0.48%). On the other hand, the magnitude of interspecific variability was lower than intraspecific variability in some cases. For example, the difference between the two most closely related species *S. bisexualis* D.G. Mann et K.M. Evans and *S. pupula* (Kützing) Mereschkowsky agg. ‘upland elliptical’ was by 4 or 5 bp (0.32–0.40%) for *rbc*L. At the same time, the authors point out the sequence divergence between the clades are similar to differences found between closely related *Sellaphora* species and constitute 0.6–2.8% (7–35 bp). To confirm the species limits, crossbreeding experiments were conducted in this work. As a result, not a single certain successful interlineage cross was observed, whereas the strains from the same lineage vigorously and successfully crossed with zygote formation.

In several studies dedicated to the *Pinnularia subgibba* Krammer group [50,51,52], it was shown that the *rbc*L and 18S rRNA genes were relatively conservative in the genus *Pinnularia*. Thus, closely related species *P*. cf. *parvulissima* Krammer and *P. lacustrigibba* Poulícková, D.G.Mann et Kollár differed only by 0.4% (6 bp) based on the comparison of the full *rbc*L sequence (1365 bp length), and by 0.5% (2 bp) based on the V4 18S rDNA region (397 bp). The genetic distance of the *rbc*L sequence between other closely related *Pinnularia* species was 0.36–0.73% (or 5–10 bp) and 0.5–1% (or 2–4 bp) for the V4 18S rDNA region.

In a study dedicated to cryptic diversity in the terrestrial diatom *Pinnularia borealis* Ehrenberg, the most variable marker was the nuclear encoded 28S gene [34]. The maximum difference between strains was 114 bp (18.8%), whereas this variation was much lower for *rbc*L with a maximum sequence divergence of 43 bp (3.5%). The two most closely related lineages of *Pinnularia borealis* differed from each other by 9–14 bp (0.7–1.0%) for *rbc*L. Interestingly, detailed morphological examinations in LM and SEM could not reveal conclusive differentiation among several closely and distantly related lineages in the *P. borealis* species complex.

According to Pinseel et al. [35], the *rbc*L gene was the most variable marker for the *Achnanthidium minutissimum* (Kützing) Czarnecki species complex. The authors found 12 different phylogenetic lineages. Strains differed from each other by 0–63 bp (0–4.4%) with a mean of 25.2 bp (2.0%). For the nuclear encoded 18S gene, the genetic distance was 0–22 bp (0.0–4.2%) with a mean of 8.7 bp (1.1%). However, some strains showed small but consistent morphological differences revealing two distinct groups, and both morphological sublineages had almost identical sequences for *rbc*L (3–4 bp difference) and showed no difference in 28S and 18S.

Thus, it can be concluded that low genetic distance (0.1–0.7%) for *rbc*L and 18S rDNA regions is often observed between closely related diatom species. However, it is not always possible to establish links between morphological variability and genetic divergence.

Our phylogenetic analysis based on the *rbc*L and V4 18S rDNA regions allowed us to confidently assign most of the strains to species (Figure 1). The species *H. cattiensis* sp. nov., *H. vietnamica* sp. nov. (VP241), *H. paravietnamica* sp. nov. (VP128), and *H. concava* (VP169), along with genetic isolation, have distinct morphological differences, on the basis of which we described them as new to science.

The strains we assigned to *H. cattiensis* sp. nov. (VP254, VP242, VP252, and VP243) have no differences in *rbc*L and V4 18S rDNA sequences among themselves and show stable morphological characteristics (valve shape is linear, slightly inflated in the center, with bluntly rounded ends, rounded central area, and hyaline areas at the ends, with a width of 2.8–3.2 µm and a striae density of 30–36 in 10 µm), separating them from other known *Humidophila* species. The genetic distance with the other species in our samples is 0.5–4.8% for *rbc*L and 1.0–5.5% for V4 18S rDNA, which, combined with morphological features, clearly separates this group from other strains (Table 6 and Table 7). Interestingly, all strains of *H. cattiensis* sp. nov. were isolated from samples collected at the same test site “Ficus” (see “Materials and Methods”) but at different soil horizons. The strain with the smaller valve length VP252 was isolated from a depth of 5–10 cm, medium-length VP254—from a fallen sediment sample, and large strains VP242, VP243—from 0 to 1 cm surface soil samples (Figure 2A–N and Figure 3).

*H. concava* sp. nov. (Figure 7) is the most unique both in morphology and phylogeny. The strain forms a separate lineage on the phylogenetic tree (Figure 1). The sequence divergence between *H. concava* sp. nov. and other species is 1.7–7.4% for V4 18S rDNA and 3.8–3.5% for *rbc*L (Table 6 and Table 7).

Species *H. vietnamica* sp. nov. and *H. paravietnamica* sp. nov. are each represented by a single strain (VP241 and VP128, respectively) and form a single lineage on the phylogenetic tree. The species are very similar in size but clearly differ in valve outline (Figure 5A–N and Figure 6A–M) and ultrastructure (elongated striae on the outer valve surface in *H. vietnamica* sp. nov., which are absent in *H. paravietnamica* sp. nov.) (Figure 5O and Figure 6N). Genetically, the species are separated by both marker genes. Sequence differences between species are 2.0% (7 bp) for V4 18S rDNA and 0.9% (or 13 bp) for *rbc*L, and, with the rest of *Humidophila*, 1.6–7.0% for V4 18S rDNA and 0.7–4.9% for *rbc*L. All this confirms the species separation.

Strain VP110 of *Humidophila* “*bacilliformis*” morphotype forms a separate lineage on the phylogenetic tree with maximum support (BS 99; PP 1.0). The analysis of variability of genetic markers revealed some peculiarities. Strain VP110 is practically identical to the group of strains of subclade A by *rbc*L. The differences are only 0.2% (or 2–3 bp), with 100% similarity in amino acids. The genetic distance with other strains of the genus is 0.1–4.8% (Table 6, Table 7 and Table 8). Significant differences in this strain from other strains were noted in the V4 18S rDNA region—4.4–7.4%. At the same time, this strain is very close in morphology to several strains of the morphotype “*bacilliformis*” from subclade A, namely small-celled VP111, VP114, VP161, VP244, and VP120) (Figure 8, Table 3). Thus, while there is a distinct genetic differentiation of strain VP110, we did not find clear morphological and morphometric characters (features), separating this strain from the above-mentioned strains. We can assume that this strain (or strains of this morphotype) was isolated at a late stage of the life cycle, when morphological features may be partially lost. However, we worked with accumulative cultures and strain VP110 was isolated from one Kt9 sample simultaneously (after one month of incubation in the laboratory) with morphologically similar but genetically distinct subclade A strains (VP108, VP112, and VP114 (Figure 8)) and with morphologically and genetically distinct strain VP119 (Figure 12). No microalgae were detected in the natural samples when viewed immediately after collection. Therefore, it is not possible to determine phenotypic variability in natural conditions and connect it to the culture material.

**Subclade A**

The set of subclade A strains is an interesting example: firstly, given the complete genetic homogeneity of *rbc*L and V4 18S rDNA marker regions (100% sequence similarity) of strains VP108, VP111, VP112, VP114, VP120, VP161, VP244, VP251, and VP 253 (Figure 1, Table 6 and Table 7), we observe clear morphological differentiation in at least two morphotypes, *H. “lanceolate-triundulate”* (strain VP253) and *H. “bacilliformis”* (strains VP108, VP112, VP114, VP161, VP251, VP120, and VP244). Secondly, strain VP119 identified by us as *H.* cf. *platensis* clearly differs in morphology from all other strains of the clade and represents a third morphotype. Differences in nucleotide sequences from the other strains of subclade A were noted only for the *rbc*L region and amounted to 0.1% (or 2 bp). At the same time, the sequences are completely identical in amino acids (Table 8). With the other species of our sample, the differences in the V4 region are 1.0–4.1% and in the *rbc*L region—0.6–4.7%. Finally, strain VP111 differs only by one deletion in the V4 region (sequences in the *rbc*L region are completely identical) and belongs to the morphotype *H.* “*bacilliformis*”.

Interestingly, the clade is composed of strains isolated from three soil samples taken from different test sites (see Materials and Methods): “Afzelia” (Kt26 (0–1 cm) strain VP161—*H.* “*bacilliformis*”), “Vyshka” (Kt9 (0–1 cm), strains VP108, VP111, VP112, VP114, VP120—*H.* “*bacilliformis*”), VP119—*H.* cf. *platensis*,), “Ficus” (Kt16 (depth 5–10 cm), strains VP244, VP251—*H.* “*bacilliformis*”, VP253—*H.* “*lanceolate-triundulate*”).

Thus, using the example of strains of this clade, we can conclude that the V4 18S rDNA and *rbc*L genetic markers selected for this study, in this case, do not allow us to distinguish morphotypes *H.* “*bacilliformis*” and *H.* “*lanceolate-triundulate*” and do not clearly separate *H.* cf. *platensis*. Therefore, further studies are needed using other markers (for example 28S rDNA regions, cox1, *psb*A), which may be more variable and allow the separation of morphotypes at the genetic level.

## 4. Materials and Methods

**Figure 15:**
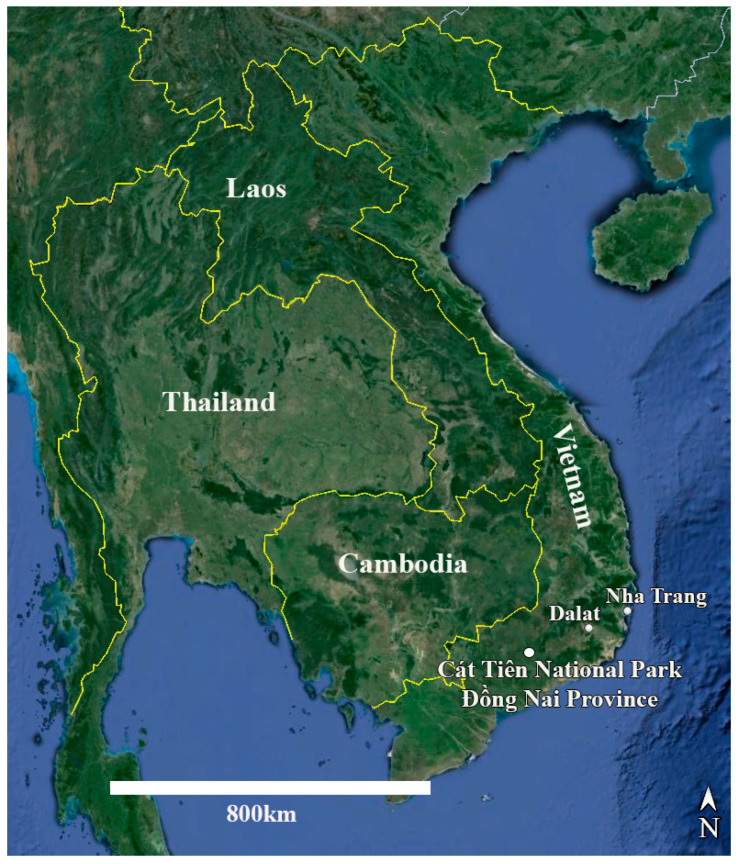
Map of Vietnam and neighboring countries with the location of Cát Tiên National Park marked.

**Study area.** Cát Tiên National Park is located in southern Vietnam, 150 km northeast of Ho Chi Minh City (Figure 15). The park was established in 1978 and consists of three parts with a total area of 73,878 ha [53,54]. The region is categorized as having the bioclimatic type of monsoon tropical climate with summer rains. The average annual temperature is about 26 °C, and relative humidity always exceeds 70%. The dry season continues from December to March, and there is almost no rainfall. The wet season continues for 8 months, from April to November, with peaks in August–September. At this time of the year, there are up to 400–450 mm of precipitation falls per month, which leads to flooding of a significant part of the park. The main part of the territory is occupied by forests, which are of the monsoon, semi-deciduous type. These forests are characterized by high biological diversity and high productivity, second only to moist tropical forests in this respect [53].

The territory of the park represents a hilly plain with absolute heights from 80 to 300 m a.s.l. It is included in the system of mountain ridges, tablelands, and intermontane valleys of the Southern Annamite Mountains. The central place in this system belongs to five ancient basaltic plateaus elevated at about 500–800 m a.s.l. and referred to as the Western Highlands (Tây Nguyên) region. Geologically, this territory is composed of slates covered by basalts. The latter have been transformed into a relatively loose material, though hard basaltic rocks are also exposed to the surface in some places. The thickness of basalts is variable, and they are often exposed to the surface as coarse rock fragments with fine earth filling spaces between them and represent the major type of soil forming rocks. In some parts, the soils are developed from clayey slates. The park is crossed by the Dong Nai River, the second largest river of southern Vietnam [55].

The several test plots on the territory of the Cát Tiên National Park were established by researchers from the Laboratory of Terrestrial and Applied Ecology of the Joint Russian-Vietnamese Tropical Research and Technology Center of the Severtsov Institute of Ecology and Evolution. The complex investigations have been conducted on these plots for many years [54,55].

**Figure 16:**
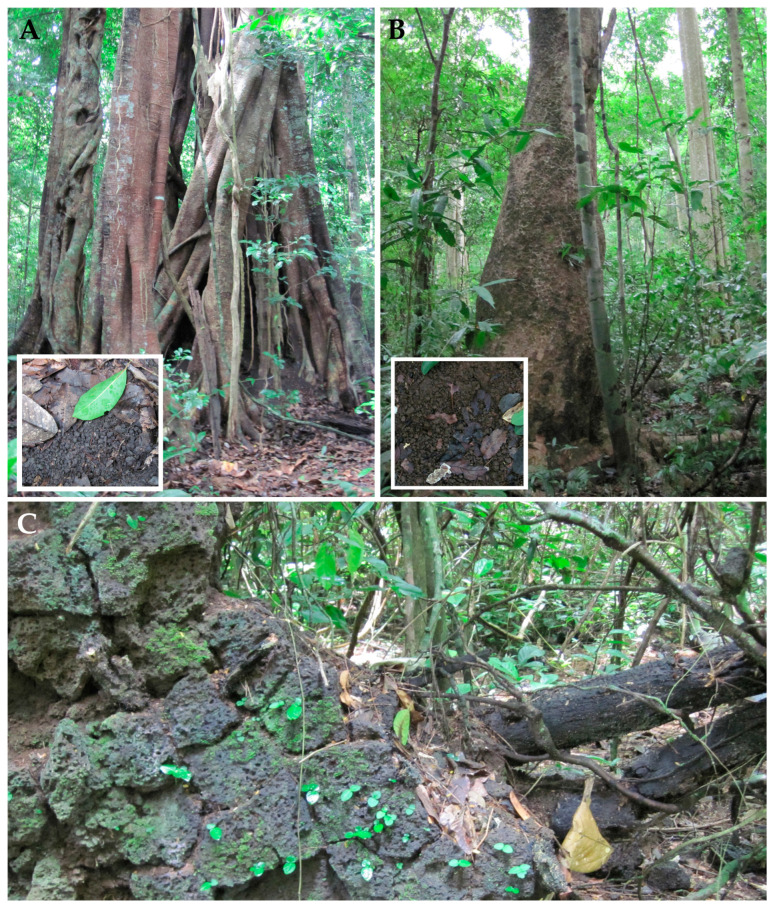
Photographs of sampling sites. (**A**) test plot “Ficus”; (**B**) test plot “Afzelia”; (**C**) basalt from which sample Kt53 was taken.

**Sampling.** Soil samples were collected by Evgeniy Gusev and Elena Kezlya in June 2019 during an expedition of the Joint Russian-Vietnamese Tropical Research and Technological Centre (the “Ecolan 1.2” Project). Sampling was carried out at 7 test plots of the National Park, but *Humidophila* taxa were found only at 3 (“Afzelia”, “Vyshka”, “Ficus”) (see Table 9 and Figure 16). Sampling was carried out from three horizons, according to standard methods: 0–5 cm, 5–10 cm, and 20–25 cm [56]. Briefly, a clean shovel was treated with alcohol, then a hole with vertical walls 30–40 cm deep was dug (depending on the depth of the underlying basalt layer). Samples (about 300 g of the soil) were placed in plastic zip bags and labeled. In order to prevent cross-contamination between samples, after taking a sample, each time the scoop was cleaned of soil residues using improvised means, it was then thoroughly wiped with a clean cloth soaked in 96% ethyl alcohol. Immediately after the sampling, the absolute humidity was determined in the laboratory room by the “hot drying” method [57], then the soil samples were air dried and packaged.

Test plots are located in forest areas and differ in soil types and higher plant communities. A brief description of the vegetation of the model sites is given using data from A.E. Anichkin [58], and descriptions of soils are given based on materials of Khokhlova et al. [55].

Test plot “Ficus” (Figure 16A) has a very gentle slope of the interfluve, closer to the divide. Almost the entire area is under the *Ficus* sp. canopy. For most of the year, the soil is covered with semi-decomposed ficus leaves. The soils are developed from the colluvium of basalts with an admixture of tuff, tephra, and fragments of volcanic bombs. These soils are characterized by the weak differentiation in color with a predominance of brownish dark gray color in the upper horizon and somewhat lighter color in the lower part of the profile. The soil texture is light clayey in the upper horizon, medium clayey in the middle part of the profile, and heavy clayey in the lower horizons. They are classified as dark clayey tropical soils developed from the derivatives of basaltic rocks.

Test plot “Afzelia” (Figure 16B) has a very gentle slope of the interfluve. The forest stand of the first sublayer is dominated by *Lagerstroemia calyculata* and *Afzelia xylocarpa*, in the second and third—the *Ficus* species and single herbs. There are more leaves of *Afzelia xylocarpa* in the leaf litter. The soils are developed from basaltic and tuff rocks with the high content of ash material; these rocks are strongly weathered. Soils are moderately acid. Particle-size distribution data indicate that the soils are light clayey in the humus horizon and heavy clayey in the lower horizons, where the content of the clay fraction is the highest. The soils are classified as thin clayey brown tropical soils.

Test plot “Vyshka” has a very gentle slope of the interfluve. The forest stand consists mainly of *Lagerstroemia calyculata*, *Ficus* sp., and *Afzelia xylocarpa*, and herbs are absent. The soil has the same characteristics as the “Afzelia” site.

Another sample used in this study (a piece of basalt) was taken from a fallen tree. (Kt53, Figure 16C).

In order to perform a quantitative analysis of the algae, the first examination of soil samples was carried out immediately after collection in the laboratory of the Joint Russian-Vietnamese Tropical Research and Technological Centre in the Cát Tiên National Park using a light microscope. The samples were examined by direct counting in a soil sample [56] (pp. 32–33). Given that sampling took place during the wet season, we expected to detect microalgae in the samples. However, the review of fresh soil samples did not reveal algae and cyanobacteria. Algae were found only after incubation of moistened soil samples in the Laboratory of Molecular Systematics of Aquatic Plants of the Institute of Plant Physiology of the Russian Academy of Sciences (Moscow, Russia). Moreover, in samples Kt9 and Kt26, microalgae were detected after about 25–30 days, while, in the other samples, the strains could be isolated only after 4 months.

**Culturing**. Gathered materials were processed in the Laboratory of Molecular Systematics of Aquatic Plants of Institute of Plant Physiology of the Russian Academy of Sciences (IPP RAS). In order to prepare cultures, the soil sample was thoroughly mixed, and a small amount (15–20 g) was placed into a Petri dish (diameter 60 mm), then saturated with distilled water up to 60–80% of full moisture capacity. Then, the Petri dish was placed into an illuminated climate chamber. After being in the chamber for 10 days, for algae detection, a little distilled water (3–5 mL) was added to the soil sample and shaken slightly, then the liquid was transferred to another Petri dish and observed with an inverted microscope Zeiss Axio Vert A1. Such observations were carried out every 10–14 days. Algal cells were extracted with a micropipette, washed in 3–5 drops of sterile distilled water, and placed into a 300 µL well on a plate for enzyme-linked immunoassay with WC liquid medium [59]. Non-axenic unialgal cultures were maintained at 22–25 °C in a growth chamber with a 12:12 h light/dark photoperiod. The strains were deposited in the Culture and Barcode Collection of Microalgae and Cyanobacteria “Algabank” (СВМС) at K.A. Timiryazev Institute of Plant Physiology RAS.

**Measurement of pH.** To measure pH, we weighed 30 g of soil to which 150 mL of distilled water was added. The suspension was poured into a clean glass, and measurements were made using the Hanna Combo device (HI 98129; Hanna Instruments, Inc., Woonsocket, RI, USA) [60].

**Preparation of slides and microscope investigation**. The culture was treated with 10% hydrochloric acid to remove carbonates and washed several times with deionized water for 12 h. Afterwards, the sample was boiled in concentrated hydrogen peroxide (≈37%) to remove organic matter. It was washed again with deionized water four times at 12 h intervals. After decanting and filling with deionized water up to 100 mL, the suspension was pipetted onto coverslips and left to dry at room temperature. Permanent diatom preparations were mounted in Naphrax. Light microscopic (LM) observations were performed with a Zeiss Axio Scope A1 microscope (Carl Zeiss Microscopy GmbH, Gottingen, Germany) equipped with an oil immersion objective (×100, n.a. 1.4, differential interference contrast) and Axiocam ERc 5s camera (Carl Zeiss Microscopy GmbH). Valve ultrastructure was examined using a scanning electron microscope JSM-6510LV (IBIW; Institute for Biology of Inland Waters RAS, Borok, Russia) and Tescan Vega3 (Centre of Electron Microscopy of Paleontological Institute RAS). For scanning electron microscopy (SEM), part of the suspensions was fixed on aluminum stubs after airdrying. The stubs were sputter-coated with 50 nm of Au using an Eiko IB 3 machine (Eiko Engineering Co. Ltd., Tokyo, Japan). The suspension and slides are deposited in the Herbarium of K.A. Timiryazev Institute of Plant Physiology, Russian Academy of Sciences (HD), Moscow, Russia.

Diatom terminology on valve outline and raphe structures followed [61,62,63].

**Molecular Study.** Genomic DNA of the studied diatom strains was extracted from fresh cultures by Chelex 100 Chelating Resin (Bio-Rad Laboratories, Hercules, CA, USA) using protocol 2.2. Nuclear gene 18S rRNA and plastid *rbc*L gene were amplified. For the highly variable V4 region of 18S rDNA (353–390 bp), D512for and D978rev primers were used [64]. The plastid *rbc*L (962–1086 bp) was amplified using rbcL404+ [65] and dp7- [66] primers. PCR amplifications were performed using premade mastermixes (ScreenMix, Evrogen, Moscow, Russia). The amplification of the 18S rDNA was performed using the following program: 5 min of denaturation at 95 °C; followed by 35 cycles of denaturation at 94 °C (30 s), annealing at 52 °C (30 s), and elongation at 72 °C (50 s); and a final extension at 72 °C (7 min), subsequently held at 12 °C. The amplification of the *rbc*L gene was performed using the following program: 4 min of denaturation at 94 °C; followed by 44 cycles of denaturation at 94 °C (50 s), annealing at 53 °C (50 s), and elongation at 72 °C (80 s); and a final extension at 72 °C (10 min), subsequently held at 12 °C.

The PCR products were visualized on a 1.0% agarose gel stained with SYBR^TM^ Safe (Life Technologies, Carlsbad, CA, USA) and then purified using a mixture of FastAP, 10× FastAP Buffer, Exonuclease I (Thermo Fisher Scientific, Waltham, MA, USA), and water. The purified PCR products were sequenced by Sanger Sequencing method using a Genetic Analyzer 3500 instrument (Applied Biosystems, Waltham, MA, USA).

Newly obtained sequences were manually edited in Ridom TraceEdit ver. 1.1.0 (Ridom GmbH, Münster, Germany) and Mega ver. 7 software [67]. For two-gene analysis, the reads were combined with GenBank-extracted sequences of 25 diatom species including nine *Diploneis* Ehrenberg ex Cleve strains as out-group (taxa names and Accession Numbers are given in Figure 1). The 18S rDNA and *rbc*L sequences were aligned separately using the G-INS-i algorithm in the Mafft ver. 7 software (RIMD, Osaka, Japan) [68]. The resulting data set comprised 396 nucleotide sites of nuclear 18S rDNA, and 1086 sites of plastid *rbc*L regions. After the removal of the unpaired regions, the aligned 18S rRNA gene sequences were combined with the *rbc*L gene sequences into a single matrix for concatenated *rbc*L and 18S rDNA tree.

The Bayesian inference (BI) method was performed to infer the phylogenetic position of new diatom strains using Beast ver. 1.10.1 software (BEAST Developers, Auckland, New Zealand) [69]. The most appropriate partition-specific substitution models, shape parameter α, and a proportion of invariable sites (pinvar) were recognized by the Bayesian information criterion (BIC) in jModel-Test ver. 2.1.10 software (Vigo, Spain) [70]. This BIC-based model selection procedure selected the following models, shape parameter α, and a proportion of invariable sites (pinvar): TrN + I+G, α = 0.5520 and pinvar = 0.4590 for 18S rDNA; TPM1uf + I+G, α = 0.6040 and pinvar = 0.6880 for the first codon position of the *rbc*L gene; JC + I, pinvar = 0.9110 for the second codon position of the *rbc*L gene; GTR + I+G, α = 3.0110 and pinvar = 0.3700 for the third codon position of the *rbc*L gene. However, the HKY model was applied instead of TPM1uf, the F81 applied instead of JC, and the GTR applied instead of TrN as the most similar applicable options for BI. A speciation model was performed by a Yule process tree prior. Five MCMC analyses were run for seven million generations (burn-in 1000 million generations). The convergence diagnostics was performed in the Tracer ver. 1.7.1 software (MCMC Trace Analysis Tool, Edinburgh, UK) [69]. The initial 15% trees were removed, with the rest retained to construct a final chronogram with 90% posterior probabilities. The robustness of tree topologies was assessed by boot-strapping the data set with maximum likelihood (ML) analysis using RAxML software (https://github.com/stamatak/standard-RAxML, access on 23 March 2025) [71]. The ML bootstrapping was performed with 1000 replicas. Trees were viewed and edited using FigTree ver. 1.4.4 (University of Edinburgh, Edinburgh, UK) and Adobe Photoshop CC ver. 19.0 software.

The 18S rDNA and *rbc*L sequences were also used to estimate the degree of similarity between gene sequences of different *Humidophila* strains. Using Mega7 software, the p-distances were determined to calculate the sequence similarity with the formula (1–p) × 100.

## 5. Conclusions

Cases of genetic variability in morphological homogeneity or cryptic diversity (in our study, this is exemplified by the *H.* “*bacilliformis*” morphotype) are frequently reported both among diatoms [34,72,73,74,75] and in other groups of microalgae, e.g., green algae [76,77,78,79], red algae [80,81], euglenoids [82,83,84], etc. These cases are probably related to the different rates of morphological and molecular evolution [85] or parallel/convergent evolution (adaptive or neutral) in which similar morphological variations might be generated in distantly related lineages [34].

Presently, there are no definite conclusions about the relationship of phylotypes in microalgae with geographic distribution or the ecological preferences. In studies investigating cryptic diversity of common taxa, both cosmopolitan and restricted geographic distributions have been shown for phylogenetic lineages [34,72,75,86], with distinguishing and overlapping ecological preferences among genetic variants [87].

The opposite cases, when different morphotypes have the same sequences of genetic markers (in our study it is Clade A, the morphotypes *H. “lanceolate-triundulate”*, *H. “bacilliformis”*, *H.* cf. *platensis*), are connected with insufficient variability for discrimination between closely related species. Although the markers 18S and *rbc*L that were selected for the present study are generally considered to be good markers for species level in diatoms [48,64,75], studies of closely related species (e.g., [34,35,51] and this study) have shown these markers to have insufficient resolution.

In this study, we examined 18 strains of *Humidophila*. These are small-celled species with sparse morphology, the differences between which can only be accurately established by SEM. Molecular data from the V4 18S rDNA and *rbc*L regions clearly confirmed the distinction of four new species from the seven morphotypes studied. Further studies of *Humidophila* are needed to properly understand the relationships between morphology, molecular data, ecological preferences, and geographical distribution.

## Figures and Tables

**Table 1:** Main morphometric features of *H. cattiensis* sp. nov. and similar taxa.

Taxon (Strain)	Length (µm)	Width (µm)	Striae Density in 10 µm	Features	Reference
*H. cattiensis* sp. nov. (VP242, VP243, VP252, VP254)	17.5–26.0	2.8–3.3	30–36		this study
*H. vietnamica* sp. nov. (VP241)	25.0–25.5	3.0–3.5	34–36	Elongated areolae at the apices	this study
*H. paravietnamica* sp. nov. (VP128)	24.5–26.0	2.5–3.0	33–35		this study
*H. subtropica*	12.0–18.0 (type) 10.0–17.0 [1]	2.7–3.5 (type) 2.3–2.5 [1]	35–40		[1,36] (p. 47; pl. 56, Figures 25–31)
*H. pantropica*	20.0–35.0 (type) 17.0–22.0 [1]	3.0–4.0 2.8–3.3	25–27 28–30		[1,37]
*H. australoshetlandica*	11.6–24.5	3.6–4.3	31–32	Internally a well-developed central nodule is present	[15]
*H. costei*	19–23.5	3.5–4.5	34–36	Hyaline area at the apices absent	[38] (p. 220)

**Table 6:** Percent divergence (p-distance) matrix of 23 strains on the basis of the partial 18S rRNA gene including the V4 barcoding subregion (396 bp).

	Strain	1	2	3	4	5	6	7	8	9	10	11	12	13	14	15	16	17	18	19	20	21	22	23
1	*H. cattiensis* VP242	–																						
2	*H. cattiensis* VP243	0.0	–																					
3	*H. cattiensis* VP252	0.0	0.0	–																				
4	*H. cattiensis* VP254	0.0	0.0	0.0	–																			
5	*H. paravietnamica* VP128	2.6	1.7	2.6	2.6	–																		
6	*H. vietnamica* VP241	2.3	1.6	2.3	2.3	2.0	–																	
7	*H. concava* VP169	3.4	2.6	3.4	3.4	2.9	3.7	–																
8	*H. frolihiensis* B360	4.1	3.4	4.1	4.1	3.7	4.1	3.1	–															
9	*H.* cf. *platensis* VP119	1.3	1.0	1.3	1.3	2.8	2.6	3.4	4.1	–														
10	*H. “lanceolate-triundulate”* VP253	1.3	1.0	1.3	1.3	2.8	2.6	3.4	4.1	0.0	–													
11	*H. “bacilliformis”* VP110	5.5	5.3	5.5	5.5	7.0	6.3	7.4	8.5	4.6	4.6	–												
12	*H. “bacilliformis”* VP108	1.0	1.0	1.0	1.0	1.4	1.6	2.6	3.1	0.0	0.0	4.5	–											
13	*H. “bacilliformis”* VP111	1.3	1.0	1.3	1.3	2.9	2.6	3.4	4.1	0.0	0.0	4.4	0.0	–										
14	*H. “bacilliformis”* VP112	1.3	1.0	1.3	1.3	2.8	2.6	3.4	4.1	0.0	0.0	4.6	0.0	0.0	–									
15	*H. “bacilliformis”* VP114	1.3	1.0	1.3	1.3	2.8	2.6	3.4	4.1	0.0	0.0	4.6	0.0	0.0	0.0	–								
16	*H. “bacilliformis”* VP120	1.3	1.0	1.3	1.3	2.8	2.6	3.4	4.1	0.0	0.0	4.6	0.0	0.0	0.0	0.0	–							
17	*H. “bacilliformis”* VP161	1.3	1.0	1.3	1.3	2.8	2.6	3.4	4.1	0.0	0.0	4.6	0.0	0.0	0.0	0.0	0.0	–						
18	*H. “bacilliformis”* VP244	1.3	1.0	1.3	1.3	2.8	2.6	3.4	4.1	0.0	0.0	4.6	0.0	0.0	0.0	0.0	0.0	0.0	–					
19	*H. “bacilliformis”* VP251	1.3	1.0	1.3	1.3	2.8	2.6	3.4	4.1	0.0	0.0	4.6	0.0	0.0	0.0	0.0	0.0	0.0	0.0	–				
20	*H. sceppacuerciae* D300_002	3.9	3.1	3.9	3.9	2.8	3.3	1.7	3.3	3.3	3.3	7.1	2.4	3.3	3.3	3.3	3.3	3.3	3.3	3.3	–			
21	*H. sceppacuerciae* D300_022	3.9	3.1	3.9	3.9	2.8	3.3	1.7	3.3	3.3	3.3	7.1	2.4	3.3	3.3	3.3	3.3	3.3	3.3	3.3	0.0	–		
22	*Nupela indonesica* Ind121	3.6	3.6	3.6	3.6	3.3	3.6	1.9	4.2	3.6	3.6	7.8	3.3	3.6	3.6	3.6	3.6	3.6	3.6	3.6	2.6	2.6	–	
23	*Nupela lesothensis* Ind168	4.9	3.9	4.9	4.9	4.3	4.1	4.3	4.9	5.1	5.1	8.5	4.2	5.2	5.1	5.1	5.1	5.1	5.1	5.1	3.6	3.6	2.6	–

**Table 7:** Percent divergence (p-distance) matrix of 23 strains on the basis of the *rbc*L gene (1086 bp).

	Strain	1	2	3	4	5	6	7	8	9	10	11	12	13	14	15	16	17	18	19	20	21	22	23
1	*H. cattiensis* VP242	–																						
2	*H. cattiensis* VP243	0.0	–																					
3	*H. cattiensis* VP252	0.0	0.0	–																				
4	*H. cattiensis* VP254	0.0	0.0	0.0	–																			
5	*H. paravietnamica* VP128	0.7	0.7	0.7	0.7	–																		
6	*H. vietnamica* VP241	0.7	0.7	0.7	0.7	0.9	–																	
7	*H. concava* VP169	4.6	4.6	4.6	4.6	4.4	4.6	–																
8	*H. frolihiensis* B360	4.8	4.8	4.8	4.8	4.9	4.9	5.5	–															
9	*H.* cf. *platensis* VP119	0.6	0.6	0.6	0.6	0.7	0.9	4.3	4.7	–														
10	*H. “lanceolate-triundulate”* VP253	0.5	0.5	0.5	0.5	0.7	0.8	4.5	4.9	0.1	–													
11	*H. “bacilliformis”* VP110	0.7	0.7	0.7	0.7	0.8	1.0	4.4	4.8	0.1	0.2	–												
12	*H. “bacilliformis”* VP108	0.5	0.5	0.5	0.5	0.7	0.8	4.5	4.9	0.1	0.0	0.2	–											
13	*H. “bacilliformis”* VP111	0.5	0.5	0.5	0.5	0.7	0.8	4.5	4.9	0.1	0.0	0.2	0.0	–										
14	*H. “bacilliformis”* VP112	0.5	0.5	0.5	0.5	0.7	0.8	4.5	4.9	0.1	0.0	0.2	0.0	0.0	–									
15	*H. “bacilliformis”* VP114	0.5	0.5	0.5	0.5	0.7	0.8	4.5	4.9	0.1	0.0	0.2	0.0	0.0	0.0	–								
16	*H. “bacilliformis”* VP120	0.5	0.5	0.5	0.5	0.7	0.8	4.5	4.9	0.1	0.0	0.2	0.0	0.0	0.0	0.0	–							
17	*H. “bacilliformis”* VP161	0.5	0.5	0.5	0.5	0.7	0.8	4.5	4.9	0.1	0.0	0.2	0.0	0.0	0.0	0.0	0.0	–						
18	*H. “bacilliformis”* VP244	0.5	0.5	0.5	0.5	0.7	0.8	4.5	4.9	0.1	0.0	0.2	0.0	0.0	0.0	0.0	0.0	0.0	–					
19	*H. “bacilliformis”* VP251	0.5	0.5	0.5	0.5	0.7	0.8	4.5	4.9	0.1	0.0	0.2	0.0	0.0	0.0	0.0	0.0	0.0	0.0	–				
20	*H. sceppacuerciae* D300_002	4.4	4.4	4.4	4.4	4.4	4.3	3.8	5.2	4.5	4.6	4.6	4.6	4.6	4.6	4.6	4.6	4.6	4.6	4.6	–			
21	*H. sceppacuerciae* D300_022	4.4	4.4	4.4	4.4	4.4	4.3	3.8	5.2	4.5	4.6	4.6	4.6	4.6	4.6	4.6	4.6	4.6	4.6	4.6	0.0.	–		
22	*Nupela indonesica* Ind121	5.6	5.6	5.6	5.6	5.6	5.5	6.7	6.4	5.5	5.7	5.6	5.7	5.7	5.7	5.7	5.7	5.7	5.7	5.7	6.3	6.3	–	
23	*Nupela lesothensis* Ind168	5.5	5.5	5.5	5.5	5.5	5.4	6.3	6.2	5.4	5.6	5.5	5.6	5.6	5.6	5.6	5.6	5.6	5.6	5.6	5.9	5.9	1.0	–

**Table 8:** Percent divergence (p-distance) matrix of 23 strains on the basis of the partial ribulose–1,5–bisphosphate carboxylase, large subunit (362 amino acids).

	Strain	1	2	3	4	5	6	7	8	9	10	11	12	13	14	15	16	17	18	19	20	21	22	23
1	*H. cattiensis* VP242	–																						
2	*H. cattiensis* VP243	0.0	–																					
3	*H. cattiensis* VP252	0.0	0.0	–																				
4	*H. cattiensis* VP254	0.0	0.0	0.0	–																			
5	*H. paravietnamica* VP128	1.1	1.2	1.1	1.1	–																		
6	*H. vietnamica* VP241	0.9	0.9	0.9	0.9	0.9	–																	
7	*H. concava* VP169	6.1	6.6	6.2	6.2	5.6	5.3	–																
8	*H. frolihiensis* B360	6.3	6.0	6.5	6.5	5.8	5.4	7.2	–															
9	*H.* cf. *platensis* VP119	0.6	0.6	0.6	0.6	0.6	0.3	5.6	5.8	–														
10	*H. “lanceolate-triundulate”* VP253	0.6	0.6	0.6	0.6	0.6	0.3	5.6	5.9	0.0	–													
11	*H. “bacilliformis”* VP110	0.6	0.6	0.6	0.6	0.6	0.3	5.6	5.8	0.0	0.0	–												
12	*H. “bacilliformis”* VP108	0.6	0.6	0.6	0.6	0.6	0.3	5.6	5.8	0.0	0.0	0.0	–											
13	*H. “bacilliformis”* VP111	0.5	0.6	0.6	0.6	0.5	0.3	5.6	5.8	0.0	0.0	0.0	0.0	–										
14	*H. “bacilliformis”* VP112	0.6	0.6	0.6	0.6	0.6	0.3	5.7	5.9	0.0	0.0	0.0	0.0	0.0	–									
15	*H. “bacilliformis”* VP114	0.6	0.6	0.6	0.6	0.6	0.3	5.7	5.9	0.0	0.0	0.0	0.0	0.0	0.0	–								
16	*H. “bacilliformis”* VP120	0.6	0.6	0.6	0.6	0.6	0.3	5.7	5.9	0.0	0.0	0.0	0.0	0.0	0.0	0.0	–							
17	*H. “bacilliformis”* VP161	0.6	0.6	0.6	0.6	0.6	0.3	5.6	5.8	0.0	0.0	0.0	0.0	0.0	0.0	0.0	0.0	–						
18	*H. “bacilliformis”* VP244	0.6	0.6	0.6	0.6	0.6	0.3	5.7	5.9	0.0	0.0	0.0	0.0	0.0	0.0	0.0	0.0	0.0	–					
19	*H. “bacilliformis”* VP251	0.6	0.6	0.6	0.6	0.6	0.3	5.7	5.9	0.0	0.0	0.0	0.0	0.0	0.0	0.0	0.0	0.0	0.0	–				
20	*H. sceppacuerciae* D300_002	4.8	5.1	4.8	4.8	4.2	4.0	4.8	6.0	4.2	4.2	4.2	4.2	4.2	4.2	4.2	4.2	4.2	4.2	4.2	–			
21	*H. sceppacuerciae* D300_022	4.8	5.1	4.8	4.8	4.2	4.0	4.8	6.0	4.2	4.2	4.2	4.2	4.2	4.2	4.2	4.2	4.2	4.2	4.2	0.0	–		
22	*Nupela indonesica* Ind121	5.7	6.0	5.8	5.8	4.8	4.7	8.9	8.1	5.1	5.2	5.1	5.2	5.1	5.2	5.2	5.2	5.1	5.2	5.2	6.5	6.5	–	
23	*Nupela lesothensis* Ind168	5.3	5.6	5.4	5.4	4.4	4.4	7.5	7.5	4.7	4.8	4.7	4.8	4.7	4.8	4.8	4.8	4.7	4.8	4.8	5.4	5.4	2.0	–

**Table 9:** List of samples and *Humidophila* strains examined in this study with geographic locality, measured ecological parameters and GenBank accession numbers.

Collection Date	Coordinates, Local Name of the Plot	Sample no., Depth (cm) or Substrate	Total Humidity (%)	pH	Species or Morphotype	Strain	Slide No.	GenBank Accession Number, *rbc*L, Partial	GenBank Accession Number, 18S rDNA, Partial
07.06.19	11° 25.664′ N 107° 25.543′ E, “Afzelia”	Kt26, 0–1	37.90	4.8	*H. “bacilliformis*” morphotype	VP161	06844	PV393023	PV387093
05.06.19	11° 26.490’N 107° 24.063’E, “Vyshka”	Kt9, 0–1	41.24	5.1	*H. “bacilliformis”* morphotype	VP108	06771	PV393015	PV387085
					*H. “bacilliformis”* morphotype	VP110	06769	PV393016	PV387086
					*H. “bacilliformis”* morphotype	VP111	06774	PV393017	PV387087
					*H. “bacilliformis”* morphotype	VP112	06775	PV393018	PV387088
					*H. “bacilliformis”* morphotype	VP114	06763	PV393019	PV387089
					*H.* cf. *platensis*	VP119	06751	PV393020	PV387090
					*H. “bacilliformis”* morphotype	VP120	06756	PV393021	PV387091
		Kt10, 3–10	40.58	5.0	*H. vietnamica* sp. nov.	VP241	06999	PV393025	PV387095
05.06.19	11° N 26.112′ 107° 25.424′ E, “Ficus”	Kt15, 0–1	36.21	5.7	*H. cattiensis* sp. nov.	VP242	07000	PV393026	PV387096
					*H. cattiensis* sp. nov.	VP243	07001	PV393027	PV387097
		Kt16, 5–10	58.90	6.0	*H. “bacilliformis”* morphotype	VP244	07002	PV393028	PV387098
					*H. “bacilliformis”* morphotype	VP251	07009	PV393029	PV387099
					*H. cattiensis* sp. nov.	VP252	07010	PV393030	PV387100
					*H. “lanceolate-triundulate”* morphotype	VP253	07011	PV393031	PV387101
		Kt18, leaf litter	—	—	*H. cattiensis* sp. nov.	VP254	07012	PV393032	PV387102
16.06.19	11° 26.975′ N 107° 21.462′ E	Kt53, basalt	—	—	*H. paravietnamica* sp. nov.	VP128	06766	PV393022	PV387092
					*H. concava* sp. nov.	VP169	06852	PV393024	PV387094

## Data Availability

The original contributions presented in this study are included in the article. Further inquiries can be directed to the corresponding authors.
